# Revealing the Effect of Crystalline Self-Assembled Monolayer in Biomimetic Photosynapse with Ultraviolet Light Protection Capability

**DOI:** 10.1021/acsami.4c14221

**Published:** 2024-12-05

**Authors:** Ya-Shuan Wu, Wei-Cheng Chen, Yi-Sa Lin, Cheng-Liang Liu, Yan-Cheng Lin, Wen-Chang Chen

**Affiliations:** † Department of Chemical Engineering, National Taiwan University, Taipei 10617, Taiwan; ‡ Institute of Polymer Science and Engineering, National Taiwan University, Taipei 10607, Taiwan; § Department of Materials Science and Engineering, National Taiwan University, Taipei 10617, Taiwan; ∥ Advanced Research Center of Green Materials Science and Technology, National Taiwan University, Taipei 10617, Taiwan; ⊥ Department of Chemical Engineering, National Cheng Kung University, Tainan 70101, Taiwan

**Keywords:** artificial synapses, field-effect transistors, self-assembled monolayers, miniaturization, biomimetic device

## Abstract

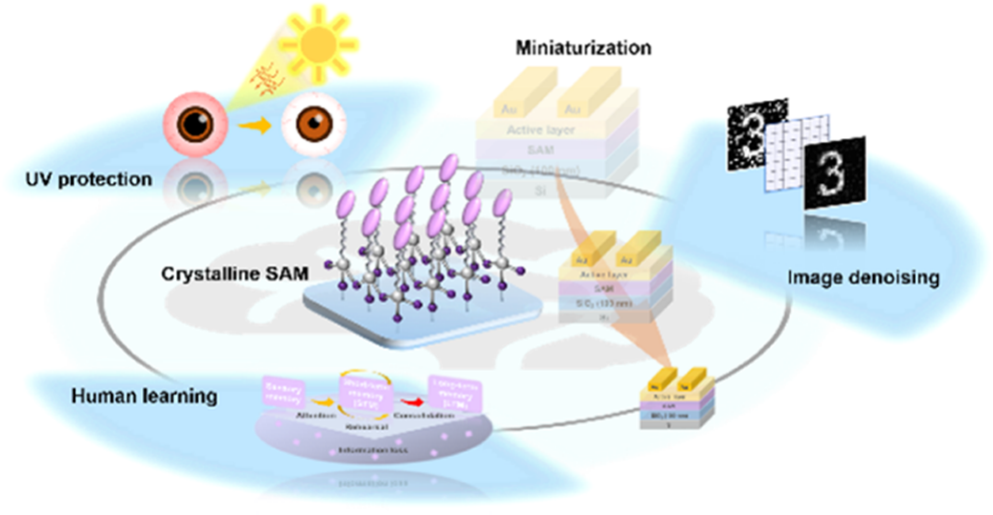

The research on photonic synapses holds immense promise for various applications, such as robotics and artificial intelligence. Pursuing lightweight, miniaturized, and low-energy consumption designs is crucial for enhancing efficiency and adaptability in evolving technological environments. To achieve this goal, this work designs a series of conjugated self-assembled molecules with photoactive pyrene, benzo-naphthol-thiophene (BNT), perylene, and benzothieno-benzothiophene cores to develop ultrathin (<3 nm) charge-trapping self-assembled monolayers (SAMs). The highly crystalline BNT forms an orderly arrangement with the semiconducting channel, further exhibiting distinguished current contrast stability (∼10^8^) and synaptic features, including paired-pulse facilitation (153%), ultralow energy consumption (28.9 aJ), and short/long-term plasticity. The device successfully demonstrates the emulation of human learning behavior and the self-protection mechanism against ultraviolet radiation utilizing crystalline and conjugated SAMs with different charge traps. Additionally, the capability of background denoising is evidenced by the high recognition accuracy (∼90%) for the preprocessed images. This study not only strengthens the diverse functionality of SAMs in optoelectronic devices but also highlights the significant potential of device miniaturization for biomimetic applications, making it a crucial contribution to the field.

## Introduction

In the period of data proliferation, the separation of memory systems and computing units in conventional von Neumann architecture leads to the lack of efficient data processing and high power consumption. Therefore, artificial synapses have been widely explored to address the limitations of the “von Neumann bottleneck” in the past few years, which provide versatile applications in image recognition, biomedical use, the Internet of Things (IoT), and robotics.^1−3^ The correlation between the human brain and artificial synapses lies in neuromorphic computing. Artificial synapses aim to emulate the visual perception system and biological function inspired by the human brain by adjusting their conductive properties after being stimulated by external stimuli, such as electrical bias and light, mimicking the synaptic plasticity in biological neurons.

Taking advantage of the tunable absorption wavelength, noncontact operation, and high transmission speed, optoelectronic devices integrate the electronic and optical functionalities and reduce the data latency and energy demand compared to conventional electrical-driven devices. In addition, approximately 80% of the external information humans receive is acquired through vision,^4^ and the retina is a fundamental cognition system of light signal; therefore, photosynaptic transistors are pivotal in emulating biological function and connecting the visual system with the human brain. To realize a high-performance photosynaptic device, advanced neuromorphic materials should be developed to exhibit high-performance synaptic behavior, such as paired-pulse facilitation/depression (PPF/PPD), excitatory/inhibitory postsynaptic currents (EPSC/IPSC), and the transition between short-term plasticity (STP) and long-term plasticity (LTP).

The charge-trapping materials used in photosynaptic devices can be categorized into three types: (i) conjugated polymers/molecules,^5−8^ (ii) metal oxide nanomaterials,^9−12^ and (iii) organic/inorganic perovskite (PVSK) or quantum dot nanocomposites.^13−17^ For instance, Jiang et al. utilized a conjugated polymer blend to fabricate an ultrabroadband optoelectronic synapse. The device can be operated with the spectral range of light from deep ultraviolet to near-infrared due to appropriate energy level alignment. With the merit of organic materials’ inherent flexibility, the proposed device’s synaptic behavior remains stable under various radii of curvature and different bending cycles, which holds significant potential in developing wearable devices.^18^ Recently, Han et al. combined photoelectricity and ferroelectricity using halide perovskite/poly(vinylidene fluoride-trifluoroethylene) (P(VDF-TrFE)) composite material, demonstrating the photoferroelectricity property. It turns out that P(VDF-TrFE) alters the PVSK crystallization kinetics to improve the PVSK film quality. Additionally, the polarization of P(VDF-TrFE) effectively enhances the photogenerated charge separation and leads to a higher photocurrent. The widely recognized classical conditioning experiment regarding Pavlov’s dog can be further performed with the photoferroelectric material, which adequately illustrates the biological learning behavior.^19^

The pursuit of device miniaturization is a crucial target in contemporary technological development, particularly within wearable medical devices and the IoT. This trend is underscored by the benefits of lightweight and increased functional density, which are essential for enhancing device performance and capabilities to mitigate the limitation of memory walls. However, in the systems of the above-mentioned charge-trapping materials, device miniaturization is challenging to realize owing to the formation of films with large thicknesses. In addition, the complicated fabrication process may lead to higher costs for mass production. Thus, this ongoing trend highlights the substantial potential of self-assembled monolayers (SAMs) in developing future technologies. SAMs can autonomously organize into highly ordered structures at the nanoscale and produce ultrathin layer thickness, effectively minimizing the device dimensions and improving the performance, simplifying the fabrication process, and reducing the cost.^20,21^ SAMs are also renowned for their surface modification and defect passivation functions.^22−24^ In a recent study, Guo et al. utilized 12-cyclohexyl dodecyl-phosphonic acid to tune the surface energy and thus improve the growth of the semiconducting layer, exhibiting both volatile and nonvolatile characteristics caused by the grain boundaries and the semiconductor/dielectric interface.^25^ In addition, the structure of SAMs by molecular design strategy can affect the device performance. However, using SAMs as charge-trapping materials in photosynaptic transistors has not been extensively investigated. The relationship between SAM’s crystallinity and charge trap types is also ambiguous.

Herein, a series of silane-based conjugated molecules with photoactive cores were designed and synthesized to fabricate the photosynaptic transistors with ultrathin (<3 nm) charge-trapping layers, including pyrene (Py), benzo-naphtho-thiophene (BNT), perylene (Pe), and benzothieno-benzothiophene (BTBT). Notably, the triethoxysilyl groups located at the SAMs’ heads experienced hydrolysis to form silanol groups, which further reacted via condensation with the hydroxy groups on the SiO_2_ (100 nm)/Si substrate, and the resulting siloxane bonds facilitated the orderly arrangement of the conjugated SAMs on the substrate surface. First, the growth of SAMs was characterized using X-ray photoelectron spectroscopy (XPS), atomic force microscopy (AFM), and time-of-flight secondary ion mass spectrometry (ToF-SIMS). The results show that different terminal conjugated cores influenced the crystallization and alignments of 2-decyl-7-phenyl[1]benzothieno[3,2-*b*][1]benzothiophene (Ph-BTBT-C10), as evidenced by grazing incidence wide-angle X-ray (GIWAXS). Accordingly, photosynaptic transistor based on BNT with superior crystallinity exhibits an impressive PPF ratio (153%) and ultralow energy consumption (28.9 aJ), which is much lower than that of a single synaptic event within the human brain (1–100 fJ).^26−28^ Furthermore, manipulating different light stimuli demonstrated the transition from short-term memory (STM) to long-term memory (LTM). In addition to emulating human learning behavior, the BNT-implemented photosynaptic transistor presents a self-protection mechanism against UV radiation through desensitization. The image denoising function was also illustrated, and the recognition accuracy was improved to ∼90% after three-time noise reduction. This study reveals the great potential of using SAMs to improve the photosynaptic device performance and take insights into the molecular design strategy, paving the way for advancing future technologies with lightweight, low power consumption, low cost, and miniaturization. The proposed concept connects the visual system with the human brain and realizes the function of sensing, memory, and processing in a biological neuromorphic system.

## Results and Discussion

### Characterization of the Conjugated Molecules for SAMs

**Figure 1:**
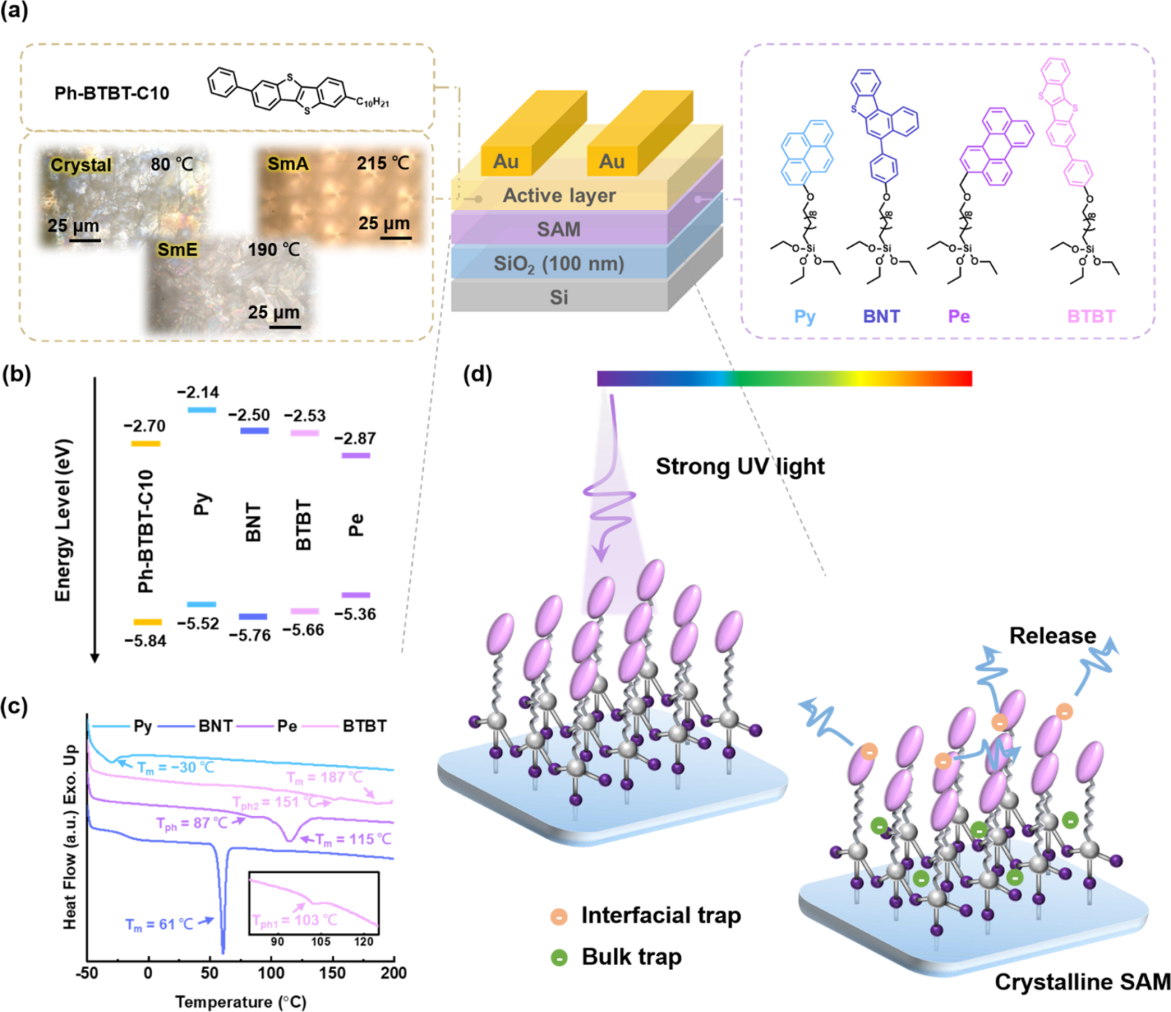
(a) Device architecture of the photosynaptic transistor, chemical structures of the semiconducting layer and the conjugated SAMs, and POM images of Ph-BTBT-C10 under different phases. (b) The frontier energy levels of constituent materials in the device. (c) Differential scanning calorimetry (DSC) heating profiles of the conjugated SAMs. (d) Schematic illustration of the self-protection mechanism of the conjugated SAMs in a photosynaptic transistor against UV radiation.

To synthesize the conjugated molecules, BNT and BTBT were first subjected to a Suzuki coupling reaction to replace the terminal groups with hydroxyl groups, and Pe was converted from an aldehyde to a hydroxyl group through a reduction reaction, facilitating subsequent alkylation reactions. The conjugated molecules were synthesized through the alkylation of conjugated cores with 10-bromo-1-decene. Then, the triethoxysilane was connected to the end of the side chain by replacing the vinyl group via hydrosilylation with Karstedt’s catalyst. The detailed synthetic procedures of the conjugated molecules for SAMs are described in the Supporting Information and Figures S1–S11 present their chemical structure characterizations. SAM-related electronic devices investigated so far are summarized in Table S1 (Supporting Information).^20,25,29−36^ Notably, the conjugated molecules in this work contribute to ultrathin SAM thickness, and introducing conjugated cores provides the photoactive and charge-trapping capability. Figure 1a illustrates the bottom-gate/top-contact (BG/TC) device architecture and the molecular structures of Ph-BTBT-C10 and SAMs. As can be seen, the SAMs were first grown on a silicon wafer with a 100 nm silicon oxide (SiO_2_) dielectric layer, followed by Ph-BTBT-C10 and Au sequentially deposited onto the SAMs as the semiconducting layer and electrodes, respectively. The corresponding energy levels of Ph-BTBT-C10 and SAMs are presented in Figure 1b and listed in Table S2 (Supporting Information), noting that the highest occupied molecular orbital (HOMO) levels were calculated from cyclic voltammetry (CV) (Figure S12, Supporting Information).

Thermal analyses were conducted to determine the fabrication conditions and investigate the conjugated molecules’ phase transition at elevated temperatures. The result shows that these molecules present good thermal stability with high degradation temperatures (>250 °C), as evidenced in Figure S13 (Supporting Information). Additionally, the DSC results in Figure 1c reveal that each molecule exhibits distinct phase transition behavior as the temperature increases. Py possesses a melting point lower than the room temperature, while BNT presents strong crystallinity. Pe and BTBT, on the other hand, display liquid crystalline (LC) behaviors. Pe and BNT are more crystalline at room temperature than Py and BTBT. These findings will further influence the alignment and crystalline behavior of the upper semiconducting layer, which is highly related to electrical performance and charge trap types. The charges are stored at the SAM/semiconductor interface for interfacial traps, while the carriers are captured in the whole SAM layer for bulk traps. Interestingly, the UV protection mechanism can be achieved when interfacial traps are more easily transferred to the semiconducting layer through the crystalline SAM/semiconductor interface, as shown in Figure 1d.

### Morphology of the Conjugated SAMs

**Figure 2:**
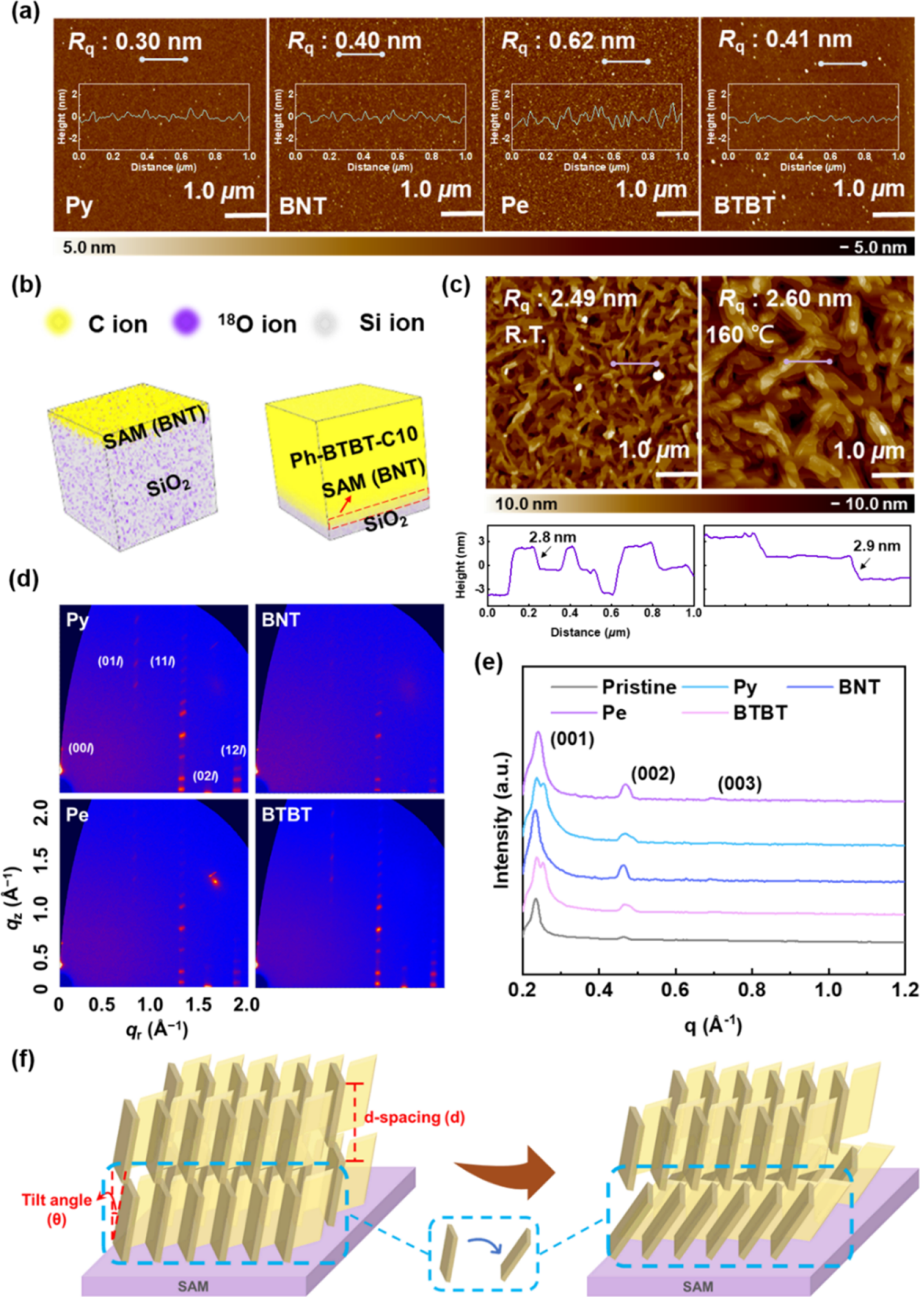
(a) AFM height images of the conjugated SAMs after thermal annealing at 160 °C. The insets are the 1D topographic profiles derived from the line-cutting in the height images. (b) ToF-SIMS 3D mappings of C, ^18^O, and Si ions for BNT/SiO_2_ and Ph-BTBT-C10/BNT/SiO_2_ structures. (c) Atomic force microscopy (AFM) height images of the Ph-BTBT-C10 layer deposited at room temperature (R.T.) or 160 °C. (d) 2D GIWAXS patterns and (e) 1D line-cutting profile in the out-of-plane direction of the Ph-BTBT-C10 deposited at 160 °C onto different conjugated SAMs. (f) Schematic illustration of the molecular alignment and tilting in Ph-BTBT-C10 standing on the crystalline SAMs (Pe, BNT; left) and amorphous SAMs (Py, BTBT; right).

After growing the SAMs on substrates, XPS analysis was conducted to confirm the formation of SAMs (Figure S14, Supporting Information). It is worth noting that the deconvolution signals of Si–O–C and Si–C bonds observed in O-1s and Si-2p bands represent their bonding with SiO_2_. As presented in Figure S15 (Supporting Information), the water contact angle measurements further show that the surfaces become more hydrophobic because of the exposed conjugated cores, which benefit the charge transfer between the conjugated SAMs and the semiconducting layer. Before the deposition of Ph-BTBT-C10, the morphologies of SAMs were verified by AFM. Figure 2a indicates that they all present relatively low surface roughness after thermal annealing compared to their initial states (Figure S16, Supporting Information), which are 0.30, 0.40, 0.62, and 0.41 nm for Py, BNT, Pe, and BTBT, respectively. The inset figures are the 1D profiles derived from the AFM topographies. In contrast to Py, BNT, and BTBT, the greater roughness found in the Pe surface can be attributed to its larger conjugated structure, leading to stronger π–π interaction and molecular aggregates. To further gain insight into the device architecture of these SAMs and the deposited Ph-BTBT-C10, ToF-SIMS analysis with 3D mappings was conducted, as provided in Figures 2b and S17 (Supporting Information). From their corresponding depth profiles (Figures S18 and S19, Supporting Information), the amount of Si ions becomes saturated within 3 nm with the decrease in C and S/^34^S ions for all the conjugated SAMs; this becomes saturated at 50 nm for the deposited Ph-BTBT-C10. This indicates the formation of an ultrathin (<3 nm) charge-trapping SAM, beneficial for the device miniaturization.

The crystallization of the semiconducting layer and the SAM/semiconductor interface significantly impact charge transfer and overall electrical characteristics. According to the previous study, Ph-BTBT-C10 was reported to undergo a phase transition as the temperature increases, and the LC behavior is shown.^37,38^ As shown in Figure 1a, the molecular alignment of Ph-BTBT-C10 was investigated using polarized optical microscopy (POM) under different temperatures. Upon heating, the initial crystalline state transitions into the smectic E (SmE) phase, which presents the fan-like texture with disclination lines, and subsequently into the smectic A (SmA) phase with focal conic texture. The SmE phase possesses good structural packing and herringbone stacking, and its end-on order enhances charge mobility;^39,40^ Thus, the Ph-BTBT-C10 was deposited on the conjugated SAMs layer under 160 °C to form the SmE phase. The surface morphology was characterized using AFM (Figures 2c and S20, Supporting Information), and the result demonstrates that the semiconducting layer deposited at 160 °C exhibits larger grain size than that deposited at room temperature, which is beneficial for homeotropic alignment and charge transport. In addition, the 1D profiles derived from AFM topographies show that the layer thickness corresponds to the molecular length of ∼2.74 nm obtained from the electrostatic potential (ESP) mapping based on the density functional theory (DFT) calculation. The molecular length and ESP mapping of Ph-BTBT-C10 were simulated using the Gaussian 09 W program with DFT, utilizing the B3LYP method with a 6-31G basis set. This finding reconfirms the end-on orientation of Ph-BTBT-C10, and the vertical alignment of molecules is conducive to forming a high-mobility channel.

After investigating the surface morphologies, the crystalline behavior within the semiconducting layer on different conjugated SAMs was further studied to understand their impact on the channel’s molecular alignment. The 2D GIWAXS patterns of the deposited Ph-BTBT-C10 under 160 °C and room temperature were presented in Figures 2d and S21a–f (Supporting Information), respectively. The diffraction peaks were labeled and revealed an enhancement of crystallinity in the case of 160 °C. The crystallite coherence length (*D*) was calculated using the Scherrer equation: *D* = 0.9 × 2π/fwhm, where fwhm is the full-width at half-maximum of (001) peak, and the lamellar spacing (*d*_001_) is determined by *d*_001_ = 2π/*q*, where *q* represents the (001) peak position. Additionally, paracrystalline disorder (*g*) and tilt angle (θ_tilt_) are estimated using *g* = (0.5 × fwhm/π/*q*)^0.5^ and θ_tilt_ = cos^–1^(*d*_001_/*L*), where *L* stands for the molecular length.^41^ The results are summarized in Tables 1 and S3 (Supporting Information). It is worth noting that under 160 °C deposition, Ph-BTBT-C10 deposited on BNT and BTBT exhibited more orderly alignments and larger crystallite sizes of 46.12 and 48.71/47.28 nm than the pristine one (40.92 nm), Py/Ph-BTBT-C10 (43.94/38.49 nm), and Pe/Ph-BTBT-C10 (39.68 nm), with Ph-BTBT-C10 on BNT being the most perpendicular (θ_tilt_ = 10.32°) to the surface. In addition, as can be observed in Figures 2e and S21g (Supporting Information), the corresponding 1D profiles along the out-of-plane direction show that Py and BTBT exhibited peak splitting at the (001) orientation under 160 °C deposition compared to room temperature, indicating a phenomenon akin to phase separation.^42^ This suggests that the Ph-BTBT-C10 molecules near the SAM/semiconductor interface are tilting on an amorphous SAM, which is detrimental to charge transport. The schematic diagram is illustrated in Figure 2f. In contrast, Ph-BTBT-C10 standing on a crystalline SAM exhibits a good end-on orientation, enhancing the electrical performance of the device. Consequently, conjugated SAMs with strong crystallinity were found to effectively improve the alignment of the semiconductor layer, thereby optimizing the device characteristics.

**Table 1:** Crystallographic Parameters Based on the (001) Diffractions, Including the Crystallite Coherence Length (*D*), *d*-Spacing (*d*_001_), Paracrystalline Disorder (*g*), and Tilt Angle of the Ph-BTBT-C10 Films Deposited at 160 °C onto Different Surfaces

	*D* (nm)	*d*_001_ (nm)	*g* (%)	tilt angle (°)
pristine	40.92	2.68	9.69	12.00
Py	43.94/38.49	2.65/2.47	9.29/9.59	14.70/25.64
BNT	46.12	2.70	9.15	10.32
Pe	39.68	2.61	9.71	17.59
BTBT	48.71/47.28	2.65/2.48	8.82/8.66	14.92/25.23

### Optical Analysis and Photophysics of Conjugated SAMs and Deposited Ph-BTBT-C10

**Figure 3:**
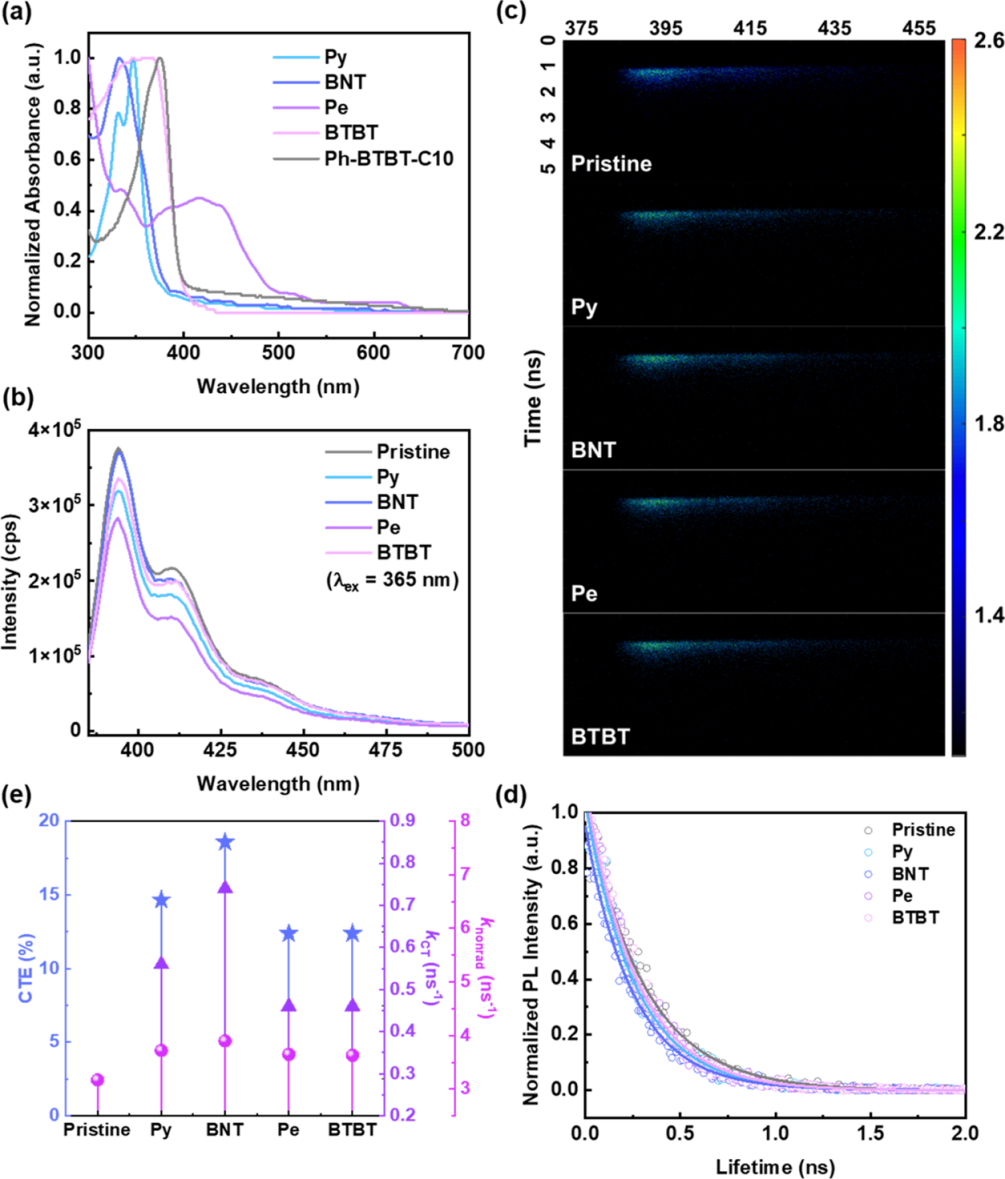
(a) UV–vis absorption spectra of the conjugated SAMs and Ph-BTBT-C10. (b) Steady-state PL emission spectra, (c) 2D TR-PL patterns, (d) 1D decay profiles, and (e) nonradiative parameters including CTE, *k*_CT_, and *k*_nonrad_ of Ph-BTBT-C10 and Ph-BTBT-C10 deposited on different conjugated SAMs. Note that the excitation wavelengths are 365 and 375 nm for the steady-state PL and TR-PL analyses, respectively.

In addition to morphologies, optical properties also play crucial roles in device performance. Thus, further optical analysis was conducted to understand the photophysical phenomena. Figure 3a provides the UV–vis absorption spectra. The result suggests that the semiconductor and the conjugated SAMs absorb UV light. Ph-BTBT-C10 presents an absorption band of 300–400 nm, while Py, BNT, and BTBT lie between 300–390, 300–385, and 300–400 nm, respectively. Pe absorbs light in the UV range and the visible spectrum, with an absorption range spanning from 300 to 500 nm, because Pe has a longer conjugation length than its analogs. Furthermore, the photoluminescence (PL) analysis of Ph-BTBT-C10 on different conjugated SAMs was carried out to investigate the recombination of photogenerated excitons. In comparison to the pristine one, the steady-state PL intensity in Figure 3b and the photoluminescence quantum yield (PLQY) of Ph-BTBT-C10 decrease when layered with different conjugated SAMs, and the corresponding PL quenching ratio and PLQY are (15.1, 2.43), (1.6, 2.45), (24.8, 1.74), and (10.8, 2.39)% for Py, BNT, Pe, and BTBT, respectively. As can be seen, relatively low values are observed in both characteristics, signifying that the exciton in this system is predominantly governed by nonradiative recombination.

Therefore, the transient photophysical properties of Ph-BTBT-C10 were further explored by time-resolved photoluminescence (TR-PL). The 2D TRPL patterns are presented in Figure 3c. The corresponding 1D decay profiles are shown in Figure 3d, fitted by the exponential function: *I*(*t*) = *I*_0_ + *A* exp(−*t*/τ), where *I* is the fluorescence intensity, *A* presents the scaling constant, and *t* stands for the fluorescence decay time. The average exciton lifetime (τ_avg_) was shortened when SAMs with different conjugated cores were grown before being deposited in the semiconducting layer. As listed in Table S4 (Supporting Information), the pristine τ_avg_ is 0.307 ns, and the lifetime reduces to 0.262, 0.250, 0.269, and 0.269 ns when layered on Py, BNT, Pe, and BTBT, respectively. The corresponding charge transfer efficiency (CTE) can be calculated using the equation of (τ_pristine_ – τ_SAM_)/τ_pristine_ × 100%, and the charge transfer rate (*k*_CT_) was estimated from 1/τ_SAM_ = 1/τ_Pristine_ + 1/τ_CT_; *k*_CT_ = 1/τ_CT_.^43^ The CTE (%) and *k*_CT_ (ns^–1^) of the bilayer systems are (14.7, 0.56), (18.6, 0.74), (12.4, 0.46), and (12.4, 0.46) for Py/Ph-BTBT-C10, BNT/Ph-BTBT-C10, Pe/Ph-BTBT-C10, and BTBT/Ph-BTBT-C10, respectively. In addition, the radiative (*k*_rad_) and nonradiative (*k*_nonrad_) recombination rates can be calculated using the formulas of 1/τ_avg_ = *k*_rad_ + *k*_nonrad_ and PLQY = *k*_rad_/(*k*_rad_ + *k*_nonrad_).^30^ The results are summarized in Figure 3e and Table S5 (Supporting Information). As can be seen, *k*_nonrad_ in all the systems is considerably larger than *k*_rad_, which again evidence that the systems in this study are primarily controlled by nonradiative recombination. Among all the SAMs, the BNT system exhibits the highest CTE and *k*_CT_, which means the more robust capability of charge transfer in BNT/Ph-BTBT-C10 than its analogs, facilitating the movement of carriers to the SAMs layer for storage. The well-performed characteristics of the BNT system may be ascribed to the following two reasons: (i) the optimal energy level alignment and (ii) a flat and clean interface that facilitates orderly alignment and heterojunction between them. First, due to the closest alignment of the HOMO and lowest unoccupied molecular orbital (LUMO) levels between BNT and Ph-BTBT-C10, this contributes to a reduction in the carrier transport barriers between these two layers, thereby enhancing the charge transfer efficiency in this system. Furthermore, since BNT molecules present a strong crystallinity, as observed in the DSC result, its orderly arrangement remarkably improves the stacking of the upper layer of Ph-BTBT-C10, enhancing the interface alignment and the carrier interactions between the two layers.

### Device Performance of the Phototransistor with a Conjugated SAM

**Figure 4:**
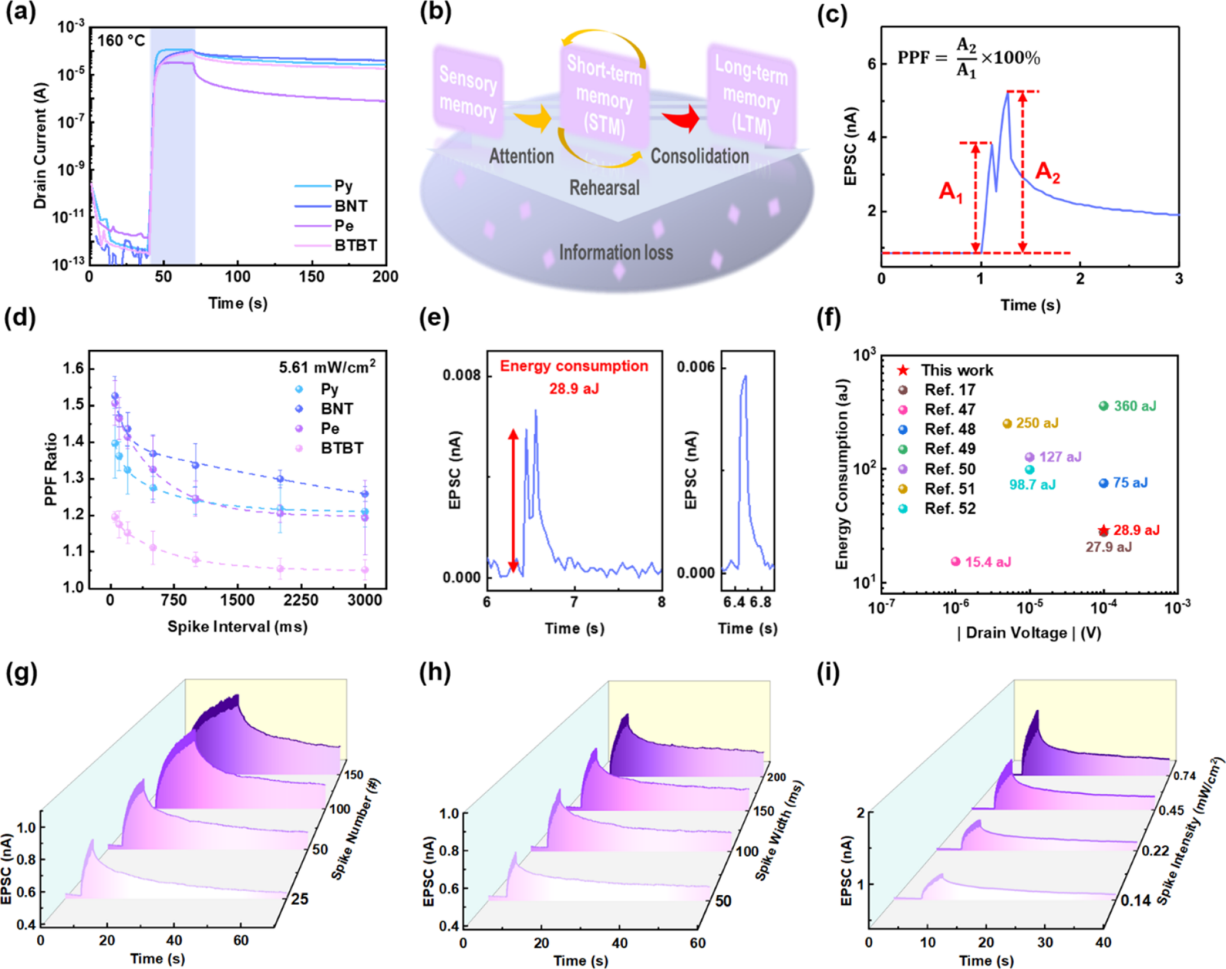
(a) Transient characteristics of conjugated SAM-based phototransistors under 365 nm-light illumination for 30 s at *V*_d_ = −50 V. (b) Schematic diagram of multistore model of memory. (c) Demonstration of consecutive spikes for PPF ratio calculation based on BNT. (d) The relationship between PPF ratios and spike intervals under 365 nm light illumination (5.61 mW/cm^2^) and a spike width of 100 ms at *V*_d_ = −3 V. (e) Energy consumption per synaptic event based on BNT under 365 nm light illumination (spike width = 50 ms; 1.53 mW/cm^2^) at *V*_d_ = −0.1 mV. (f) Summary of the energy consumption and operating voltage for the reported synaptic transistors. Emulation of human learning behavior and STM–LTM transition based on BNT, including (g) SNDP, (h) STDP, and (i) SIDP under 365 nm light illumination at *V*_d_ = −3 V. Note that the electrical properties were measured with a reading voltage of *V*_g_ = 0 V.

After examining the morphological and optical properties, the BG/TC device (Figure 1a) was fabricated to explore the LTM and STM behavior. First, the hysteresis in the SAM-based devices was verified by measuring the dual sweep transfer curves at *V*_d_ = −50 V. As can be seen in Figure S22 (Supporting Information), memory windows were generated in the devices based on Py, BNT, Pe, and BTBT with *V*_g_ sweeping forward and backward between 20 and −60 V. This indicates that the charge-trapping mechanism contributes to the hysteresis in the SAM-based phototransistors, which further provides the memory and synaptic behavior. The transfer characteristics of the SAM-based phototransistors under light illumination (365 nm; 5.61 mW/cm^2^; 30 s) and a negative bias (*V*_g_ = −60 V; 1 s) are presented in Figure S23 (Supporting Information). According to the literature, the memory behavior demonstrated in the SAM-based devices can be attributed to the molecular conformation transition under electrical and light stimuli, which provide different transport characteristics, and the contribution of structural inhomogeneity can be excluded due to the ordered array of SAMs.^44,45^ Additionally, the direct contact between the photoactive conjugated cores in SAMs and the semiconductor leads to an improved heterojunction effect, which enhances the charge-trapping capability and the charge transfer effect of photogenerated carriers using an ultrathin (<3 nm) SAM, possessing the potential in device miniaturization. A plausible mechanism for the bistable phototransistor is shown in Figure S24 (Supporting Information). Initially, the device was switched to the OFF state by applying a negative gate bias, which led to hole storage and reduced the drain current. Upon 365 nm light illumination, the trapped holes recombined with the photogenerated electrons, trapping the photogenerated electrons from Ph-BTBT-C10 of SAMs, facilitating an increase in photocurrent. The SAM-based device exhibits a bistable charge storage property, capable of trapping holes under an electrical bias while storing electrons under illumination. The proposed mechanism underlines the importance of the interaction between SAM/semiconductor interfaces. Next, the STM behavior was characterized by transient characteristics, as presented in Figure 4a. Before measuring the transient characteristics, a negative bias of *V*_g_ = −60 V was applied for 1 s at *V*_d_ = 0 V to switch the device to the OFF state. Then, the devices reached a high photoconductivity, corresponding to the ON state, under the illumination of 365 nm light (5.61 mW/cm^2^) for 30 s at a drain voltage (*V*_d_) of −50 V with a reading voltage of *V*_g_ = 0 V, which shows an exceptional ON/OFF current ratio (*I*_ON_/*I*_OFF_) of ∼10^8^ for Py, BNT, and BTBT, and ∼10^7^ for Pe. After removing the light, BNT exhibited superior current stability, while Pe displayed the fastest photocurrent decay, which stems from the alignment of SAMs with the semiconductor. Transient curves under different depositing temperatures were also measured, as shown in Figure S25 (Supporting Information). As can be seen, since Pe and BTBT show the LC behavior, devices based on them are strongly dependent on the temperature of device fabrication compared to Py and BNT-based devices, which reconfirms that the formation of SmE phase in semiconductor will lead to a preferred electrical performance.

### Emulation of the Human Learning System

The schematic diagram of the information transition after entering the human brain is illustrated in Figure 4b.^46^ First, when the brain receives external stimuli, incoming information is stored temporarily as sensory memory, then converted into STM after capturing human attention, which exists for only a few minutes. Therefore, the transition from STM to LTM requires repetitive practice. The PPF ratio is a representative parameter of STM in a photosynaptic transistor, defined as the EPSC ratio of two consecutive spikes (Figure 4c) and calculated using the equation: PPF = *A*_2_/*A*_1_ × 100%, where *A*_1_ and *A*_2_ are the first and second EPSC value. When stimulated using 365 nm light (5.61 mW/cm^2^) with a spike width of 100 ms, the PPF ratios present an exponential decay with the increasing spike interval after the conjugated SAMs were introduced as the charge-trapping layers. In contrast, the pristine one barely exhibits a memory effect under extremely brief stimuli, thus precluding its PPF capability (Figure S26a, Supporting Information). Furthermore, as can be seen in Figure S26b (Supporting Information), the EPSC of the pristine device decayed rapidly and nearly returned to its initial value after continuous light illumination (25 spikes; 0.14 mW/cm^2^; spike width = 100 ms; spike interval = 50 ms). Among all the conjugated SAMs, the highest PPF ratio of 153% was achieved by BNT (Figure 4d), and BNT also achieves a high PPF ratio of 139% at a low light intensity of 0.14 mW/cm^2^ (Figure S26c, Supporting Information). This may be due to the proper HOMO/LUMO levels and favored molecular alignment in the crystalline BNT system, making it capable of responding to a fast-trapping/detrapping mechanism. Figure S26d (Supporting Information) shows that the photoconductivity was first enhanced under illumination with 25 spikes, then gradually decayed for 15 s, known as the forgetting process. During the relearning stage, the comparable photocurrent could be achieved with only 15 spikes, implying the memory function in the photosynaptic transistor is similar to the human learning system. A photosynaptic transistor with a semiconducting layer reduced to 20 nm was further fabricated to elucidate the concept of device miniaturization. The scaled-down device based on BNT retains similar LTM and STM performances to the original one (Figure S27a–c, Supporting Information). In addition, the energy consumption (*E*_consumption_) was calculated as *E*_consumption_ = *V*_d_ × *I*_peak_ × Δ*t*, where *I*_peak_ represents the maximum EPSC, and Δ*t* is the spike width. The corresponding *E*_consumption_ under illumination (365 nm; spike width = 50 ms; 1.53 mW/cm^2^) is 28.9 aJ (Figure 4e), which is much lower than that in a biological synapse, and the *E*_consumption_s of the recently reported synaptic transistors are summarized in Figure 4f.^17,47−52^

To gain insight into the emulation of human learning behavior, Figure 4g–i demonstrate the STM–LTM transition of BNT. The learning–forgetting–relearning process of BNT is shown in Figure S26d (Supporting Information). Spike number (SNDP), spike width (STDP), and spike intensity (SIDP) were manipulated for the rehearsal process. For instance, under the illumination of 365 nm light (0.14 mW/cm^2^; spike width = 100 ms), by varying the spike number from 25–150 spikes, the maximum EPSC increased from 0.77 to 1.07 nA, and the remaining photocurrent after 40 s of light removal also improved from 0.45 to 0.62 nA. The learning behavior of the photosynaptic transistor was derived from the electron-trapping capability under light illumination, which results in a consecutive increase in photocurrent under successive light stimulation. This indicates that the STM–LTM transition in the human brain can be regulated when different parameters of external stimuli are received, having the ability to store multilevel data.

### Self-Protection Mechanism against UV Radiation

**Figure 5:**
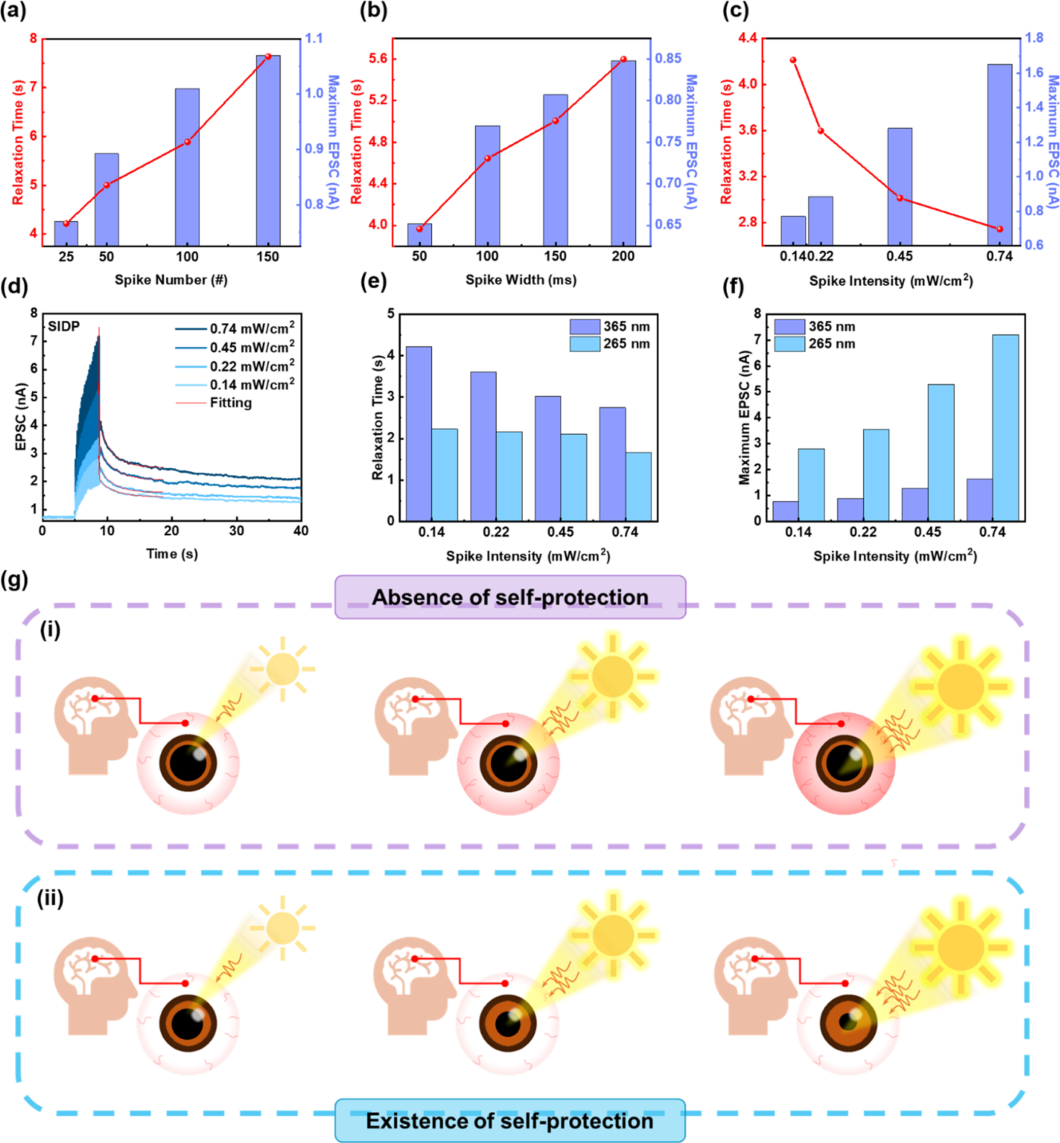
Relationship between relaxation time/maximum EPSC and (a) spike number, (b) spike width, and (c) spike intensity under 365 nm light illumination for SNDP, STDP, and SIDP, respectively. (d) STM–LTM transition of SIDP under 265 nm light illumination at *V*_d_ = −3 V. Comparison of (e) relaxation time and (f) maximum EPSC under 265 and 365 nm light illuminations with varying spike intensity (0.14–0.74 mW/cm^2^). (g) Schematic diagram of pupil regulation (part (i)) without or (part (ii)) with self-protection against UV radiation. Note that the electrical properties in (a–f) are based on the BNT-based photosynaptic device with a reading voltage of *V*_g_ = 0 V.

To understand the relationship between memory level and the amount/strength of the incoming information after stimulation, the forgetting curves in Figures 4g–i were fitted by modified Ebbinghaus’ psychological forgetting curve: *P* = Δ*G*(*t*)/Δ*G*_max_ = *B*_STM_ exp[−(*t*/τ_STM_)^β^] + *B*_LTM_ exp[−(*t*/τ_LTM_)^β^], where *P* is the recall probability, Δ*G*(*t*) = *G*(*t*) – *G*_0_ is the conductance difference between time *t* and the initial state, Δ*G*_max_ = *G*_max_ – *G*_0_ represents the conductance difference between maximum conductance and the initial state, β is the stretching exponent ranging from 0 to 1. τ_STM_ and τ_LTM_ are the relaxation times related to STM and LTM, respectively.^53−55^ A proportional relationship was found between relaxation time (τ_avg_) and the spike number/width; however, when illuminated with stronger light intensity (0.14–0.74 mW/cm^2^), τ_avg_ shows a decreasing trend from 4.2 to 2.7 s (Figure 5a–c). This implies that the device can rapidly adjust and release charge traps in response to high-intensity stimuli as a mechanism for self-protection. To elucidate the influence of light intensity on charge trap types, the interfacial traps (*N*_i_) and bulk traps (*N*_b_) were calculated using the equations of *N*_i_ = (*C*_i_/*e*^2^) × [(SS × *e*)/(*k*_B_ × *T* × ln 10 – 1)] and *N*_b_ = *C*_i_ × Δ*V*_th_/*e*, respectively, where *C*_i_ is the capacitance of SAM, *e* represents the elementary charge, SS stands for the subthreshold swing of the transfer curve in the ON state, *k*_B_ is the Boltzmann constant, and *T* is the absolute temperature.^56−58^ The transfer characteristics based on BNT device with 365 nm light intensities of 0.74 and 5.61 mW/cm^2^ are presented in Figure S27d (Supporting Information), and the corresponding (*N*_i_, *N*_b_) are (9.0 × 10^12^ eV^–1^ cm^–2^, 1.89 × 10^12^ cm^–2^) and (1.2 × 10^13^ eV^–1^ cm^–2^, 3.23 × 10^12^ cm^–2^) for 0.74 and 5.61 mW/cm^2^ light illumination, respectively. The result indicates that the interfacial trap density increases due to the enhancement of subthreshold swing. Therefore, since the carrier-trapping capacity of the interface is weakened due to the more significant number of interfacial traps, the trapped charges can be swiftly accommodated and released. In addition to the interfacial trap density, the increasing photogenerated hole density under higher light intensity further accelerates the charge recombination.^59^ A stimulus’s energy depends not only on the light intensity but also on the wavelength. Here, devices stimulated with UVA (365 nm), UVB (310 nm), and UVC (265 nm) were further investigated to demonstrate the self-protection mechanism against UV radiation in the human brain. The STM–LTM transitions under the illumination of UVB and UVC were presented in Figure S28a (Supporting Information) and Figure 5d, respectively, and the decay curves after light illumination were fitted using a modified Ebbinghaus psychological forgetting curve. Notably, regardless of the wavelength of UV light, the maximum EPSC increases with light intensity. In contrast, the relaxation time decreases as the light intensity rises (Figures 5e,f and S28b, Supporting Information). In addition to light intensity, relaxation times calculated for different wavelengths under the same intensity were further compared. Interestingly, Figure 5e shows that radiation with higher photon energy, i.e., shorter wavelength, reduces relaxation time. The conductance of the synaptic device decreases significantly after exposure to high-intensity UV light. The origin of this phenomenon is that the increasing photon intensity and energy trigger the interfacial traps inside the conjugated SAM, and these interfacial traps are more prone to recombine with the carriers inside the channel than the bulk traps, contributing to a rapid decay in EPSC.^57^ The self-protection mechanism exhibited by the photosynaptic transistor is analogous to the brain’s pupil dilation and constriction regulation. When high-intensity light enters the eye, the excess energy could potentially harm the eyeball if a self-protection mechanism is absent (Figure 5g, part (i)). On the contrary, if the brain appropriately adjusts the size of the pupil, it can effectively reduce the damage from high-energy light by dissipating the excess energy (Figure 5g, part (ii)). As a result, the connection between the human brain and the visual system is further established by the artificial synapse in this study.

### Image Denoising and Pattern Recognition

**Figure 6:**
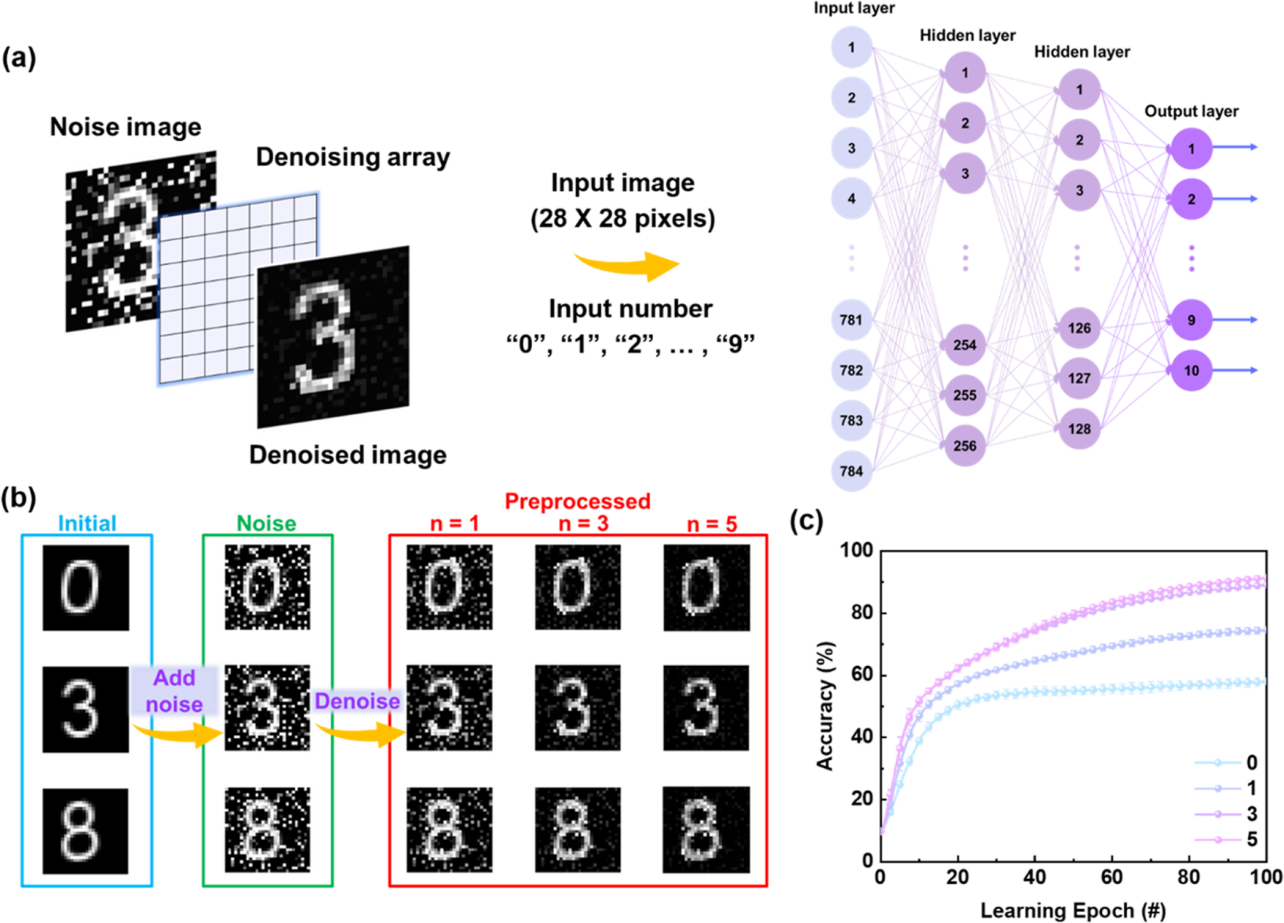
Simulation of image preprocessing: (a) schematic diagram of the image denoising process and the NN algorithm with a size of 784 × 256 × 128 × 10. (b) The results of noise adding and corresponding image denoising, and (c) recognition accuracy after repeatedly processing through the denoising array for zero, one, three, and five times.

Since image preprocessing is a crucial function of the human retina, noise reduction and image recognition were simulated with the photosynaptic transistor. The 10 000 handwritten digital images were generated from the modified National Institute of Standards and Technology (MNIST) database. Initially, random noise with a maximum noise value of 100% was added to the initial images, followed by image denoising through a denoising array, which consists of the PPF ratio obtained in the device. In the denoising array, the pixels corresponding to the digit contours were filled with the PPF values (>1), and all the other pixels were set to one, which enhanced the contrast between the background noise and the contour signals during the denoising process. Thus, a higher PPF ratio results in more effective noise reduction. To assess the performance of the denoising, the preprocessed images were further input into a four-layer neural network (NN). The schematic diagram of the artificial visual system and the architecture of the NN are illustrated in Figure 6a. Figure 6b shows that when random noise is added to the digits ‘0′, ‘3′, and ‘8′, the figures become blurred and difficult to recognize. However, after one, three, and five denoising cycles, the contours of the digits in the images become distinct, successfully removing the image background noise. Finally, the images with/without preprocessing were imported to the proposed NN to evaluate the efficacy of noise reduction. As can be seen in Figure 6c, the recognition accuracy improved significantly from 58 to ∼90% after thrice denoising and 100 learning epochs, indicating excellent noise reduction capability. The high PPF ratio enhanced the signal-to-noise ratio, and the photosynaptic transistor can realize the image preprocessing.

## Conclusions

In summary, conjugated SAMs were designed to incorporate a direct heterojunction with the semiconducting channel. The result shows that the crystallinity of SAMs leads to varying phase transition behavior, which further affects the molecular alignment and types of charge traps in the thermally deposited semiconductor. BNT exhibited remarkable current stability and higher charge transfer efficiency among all the conjugated SAMs. This can be ascribed to the following two reasons: (i) the appropriate energy HOMO/LUMO levels alignment with Ph-BTBT-C10, and (ii) strong crystallinity, which facilitates the formation of regular molecular alignment in the conjugated SAM/semiconductor interface. The crystallinity of BNT leads to a more stable charge trap owing to the higher bulk trap density with a larger memory window; on the other hand, the interfacial traps can be increased by manipulating the intensity of the light source. The synaptic behaviors were further demonstrated in BNT-based devices, including PPF ratio (153%), energy consumption (28.9 aJ) per synaptic event, and STM–LTM transition. Manipulating spike numbers, widths, and intensities can emulate human learning behavior. In addition, the self-protection mechanism against UV radiation utilizing interfacial/bulk traps was proposed, revealing that the biomimetic device can rapidly release excess energy and prevent damage from high-energy stimulation, establishing the connection between the human brain and visual perception. Finally, the simulation of image denoising was demonstrated, and a recognition accuracy of ∼90% can be reached for the preprocessed images after conducting thrice noise reduction. Through molecular design, the highly crystalline SAM with ultrathin thickness enables orderly molecular alignment, enhancing the charge transfer between SAM and semiconductor. This results in superior electrical performance and fulfills the device’s miniaturization. This study holds significant potential for future applications in synaptic devices, such as artificial intelligence and neuromorphic computing.

## Methods

### Materials

1-Pyrenemethanol (>98.0%), 5-bromobenzo[*b*]naphtho[1,2-*d*]thiophene (>97.0%), 2-bromo[1]benzothieno[3,2-*b*][1]benzothiophene (>95.0%), 10-bromo-1-decene (>95.0%), tetrabutylammonium bromide (TBAB, >98.0%), and 4-hydroxyphenylboronic acid were purchased from Tokyo Chemical Industry Co., Ltd. 3-Formylperylene was obtained from AK Scientific, Inc. Sodium hydride (NaH), tetrakis(triphenylphosphine)palladium(0) (Pd(PPh_3_)_4_), anhydrous toluene (99.8%), and *N*,*N*-dimethylformamide (DMF, 99.8%) were supplied by Sigma-Aldrich. Triethoxysilane (96%) and platinum(0)-1,3-divinyl-1,1,3,3-tetramethyldisiloxane complex solution (Karstedt’s catalyst) were purchased from Alfa Aesar. Sodium borohydride (NaBH_4_) (>98.0%) was obtained from Acros Organics. Potassium carbonate (K_2_CO_3_), anhydrous magnesium sulfate (MgSO_4_), toluene, methanol (MeOH), and hexanes were supplied by J.T. Baker Chemical Company. Tetrahydrofuran (THF) and ethyl acetate (EA) were purchased from DUKSAN. Dichloromethane (DCM) was obtained from Macron Fine Chemicals. Ph-BTBT-C10 (>99.5%) was obtained from Tokyo Chemical Industry Co., Ltd. All chemicals were used as received without further purification.

### Synthesis of 1-((Dec-9-en-1-yloxy)methyl)pyrene (Py-C10)

NaH (144 mg, 6 mmol) was first washed with hexanes, and 1-pyrene methanol (697 mg, 3 mmol) was added. Anhydrous THF (8 mL) was added under an ice bath. The reaction proceeded for 1 h under room temperature in an environment filled with Ar. After that, TBAB (96.7 mg, 0.3 mmol) was added to the solution. 10-Bromo-1-decene (0.91 mL, 4.5 mmol) was dissolved in anhydrous THF (2 mL) and then added dropwise to the solution. The reaction was conducted at room temperature for 18 h in an Ar environment and quenched with water. DCM was used to dilute the crude product, followed by sequential extractions with water and brine. The organic phase was then dried over anhydrous MgSO_4_ and subsequently filtered. The solvent was removed using a rotary evaporator. The crude product was purified by column chromatography with hexanes and DCM. Evaporation was conducted to obtain a yellow solid of 733 mg (66% yield). ^1^H NMR (500 MHz, CDCl_3_, Figure S1a), δ (ppm): 8.36–8.37 (*d*, Ar–**H**, 1H), 8.11–8.19 (*m*, Ar–**H**, 4H), 7.98–8.04 (*m*, Ar–**H**, 4H), 5.74–5.82 (*m*, CH_2_–**CH**=CH_2_, 1H), 5.20 (*s*, Ar–**CH**_**2**_–O, 2H), 4.90–4.99 (*m*, CH_2_–CH=**CH**_**2**_, 2H), 3.58–3.60 (*t*, Ar–CH_2_O–**CH**_**2**_, 2H), 1.97–2.01 (*m*, **CH**_**2**_–CH=CH_2_, 2H), 1.62–1.68 (*br*, Ar–CH_2_OCH_2_–**CH**_**2**_, 2H), 1.21–1.39 (*br*, 10H). ^13^C NMR (100 MHz, CDCl_3_, Figure S1b), δ (ppm): 26.20, 28.87, 29.04, 29.41, 29.82, 33.77, 70.55, 71.48, 114.07, 123.53, 124.46, 124.93, 125.14, 125.87, 126.88, 127.30, 127.41, 127.57 129.33, 130.83, 131.24, 131.82, 139.22.

### Synthesis of Triethoxy(10-(pyren-1-ylmethoxy)decyl)silane (Py)

A mixture of Py-C10 (500 mg, 1.35 mmol), triethoxysilane (0.5 mL, 2.70 mmol), and Karstedt’s catalyst (13.5 μL) was dissolved in anhydrous toluene (13.5 mL), and the reaction was conducted at 70 °C for 16 h in an Ar environment. DCM was used to dilute the crude product, followed by sequential extractions with water and brine. The organic phase was then dried over anhydrous MgSO_4_ and subsequently filtered. The solvent was removed using a rotary evaporator to obtain a yellow liquid of 760 mg (quantitative yield). ^1^H NMR (500 MHz, CDCl_3_, Figure S2a), δ (ppm): 8.35–8.37 (*d*, Ar–**H**, 1H), 8.11–8.19 (*m*, Ar–**H**, 4H), 7.97–8.04 (*m*, Ar–**H**, 4H), 5.20 (*s*, Ar–**CH**_**2**_–O, 2H), 3.68–3.87 (*m*, O–**CH**_**2**_–CH_3_, 6H), 3.57–3.60 (*t*, Ar–CH_2_O–**CH**_**2**_, 2H), 1.61–1.67 (*br*, Ar–CH_2_OCH_2_–**CH**_**2**_, 2H), 1.18–1.39 (*br*, 23H), 0.58–0.62 (*t*, Si–**CH**_**2**_, 2H). ^13^C NMR (100 MHz, CDCl_3_, Figure S2b), δ (ppm): 10.37, 18.01, 18.29, 29.22, 29.43, 29.60, 29.84, 33.19, 58.28, 70.61, 71.48, 123.53, 124.47, 125.12, 125.87, 126.88, 127.30, 127.42, 127.58, 129.33, 130.84, 131.25, 131.83.

### Synthesis of 4-(Benzo[*b*]naphtho[1,2-*d*]thiophen-5-yl)phenol (BNT–OH)

5-Bromobenzo[*b*]naphtho[1,2-*d*]thiophene (940 mg, 3 mmol) was dissolved in THF (10 mL), and K_2_CO_3_ (2.49 g, 18 mmol), 4-hydroxyphenyl boronic acid (828 mg, 6 mmol), and water (5 mL) were added to the solution. The mixture was freeze-pumped immediately after adding Pd(PPh_3_)_4_ (17.3 mg, 0.015 mmol). Then, the reaction proceeded at 70 °C for 48 h in an Ar environment. THF in the crude product was removed using a rotary evaporator to avoid emulsification. DCM was used to dilute the crude product, followed by sequential extractions with water and brine. The organic phase was then dried over anhydrous MgSO_4_ and subsequently filtered. The crude product was purified by column chromatography with DCM. Evaporation was conducted to give a yellow solid of 494 mg (50% yield). ^1^H NMR (500 MHz, CDCl_3_, Figure S3a), δ (ppm): 9.06–9.08 (*d*, Ar–**H**, 1H), 8.86–8.87 (*d*, Ar–**H**, 1H), 8.04–8.06 (*d*, Ar–**H**, 1H), 7.99–8.00 (*d*, Ar–**H**, 1H), 7.83 (*s*, Ar–**H**, 1H), 7.71–7.74 (*m*, Ar–**H**, 1H), 7.58–7.62 (*m*, Ar–**H**, 1H), 7.48–7.52 (*m*, Ar–**H**, 2H), 7.40–7.43 (*m*, Ar–**H**, 2H), 6.96–6.99 (*m*, Ar–**H**, 2H), 5.04 (*s*, Ar–O**H**, 1H). ^13^C NMR (100 MHz, CDCl_3_, Figure S3b), δ (ppm): 115.24, 121.89, 123.25, 123.41, 124.68, 124.88, 125.16, 126.92, 127.74, 128.38, 130.69, 131.01, 131.47, 133.01, 136.64, 138.25, 139.48, 139.75, 155.13.

### Synthesis of 5-(4-(Dec-9-en-1-yloxy)phenyl)benzo[*b*]naphtho[1,2-*d*]thiophene (BNT-C10)

BNT–OH (450 mg, 1.38 mmol) and K_2_CO_3_ (286 mg, 1.07 mmol) were dissolved in anhydrous DMF (13.8 mL). The reaction proceeded for 1 h under room temperature in an environment filled with Ar. After that, TBAB and 10-bromo-1-decene (dropwise) were added to the solution. The reaction was conducted at 60 °C for 18 h in an Ar environment. DMF in the crude product was removed using a rotary evaporator to avoid emulsification. DCM was used to dilute the crude product, followed by sequential extractions with water and brine. The organic phase was then dried over anhydrous MgSO_4_ and subsequently filtered. The solvent was removed using a rotary evaporator. The crude product was purified by column chromatography with DCM. Evaporation was conducted to obtain a yellow solid of 502 mg (78% yield). ^1^H NMR (500 MHz, CDCl_3_, Figure S4a), δ (ppm): 9.06–9.08 (*d*, Ar–**H**, 1H), 8.86–8.87 (*d*, Ar–**H**, 1H), 8.06–8.08 (*d*, Ar–**H**, 1H), 7.99–8.00 (*d*, Ar–**H**, 1H), 7.83 (*s*, Ar–**H**, 1H), 7.71–7.74 (*m*, Ar–**H**, 1H), 7.58–7.62 (*m*, Ar–**H**, 1H), 7.48–7.52 (*m*, Ar–**H**, 2H), 7.44–7.46 (*m*, Ar–**H**, 2H), 7.02–7.05 (*m*, Ar–**H**, 2H), 5.78–5.86 (*m*, CH_2_–**CH**=CH_2_, 1H), 4.92–5.02 (*m*, CH_2_–CH=**CH**_**2**_, 2H), 4.03–4.05 (*t*, Ar–O–**CH**_**2**_, 2H), 2.03–2.07 (*m*, **CH**_**2**_–CH=CH_2_, 2H), 1.81–1.86 (*br*, Ar–OCH_2_–**CH**_**2**_, 2H), 1.24–1.42 (*br*, 10H). ^13^C NMR (100 MHz, CDCl_3_, Figure S4b), δ (ppm): 26.08, 28.91, 29.43, 33.80, 68.12, 114.16, 114.34, 121.87, 123.24, 123.38, 124.66, 125.12, 126.89, 127.83, 128.30, 130.74, 131.02, 132.56, 136.67, 138.29, 139.19, 139.75, 158.74.

### Synthesis of (10-(4-(Benzo[*b*]naphtho[1,2-*d*]thiophen-5-yl)phenoxy)decyl)triethoxysilane (BNT)

A mixture of BNT-C10 (400 mg, 0.86 mmol), triethoxysilane (0.32 mL, 1.72 mmol), and Karstedt’s catalyst (8.6 μL) was dissolved in anhydrous toluene (8.6 mL), and the reaction was conducted at 70 °C for 16 h in an Ar environment. DCM was used to dilute the crude product, followed by sequential extractions with water and brine. The organic phase was then dried over anhydrous MgSO_4_ and subsequently filtered. The solvent was removed using a rotary evaporator to obtain a yellow liquid of 559 mg. ^1^H NMR (500 MHz, CDCl_3_, Figure S5a), δ (ppm): 9.06–9.08 (*d*, Ar–**H**, 1H), 8.86–8.87 (*d*, Ar–**H**, 1H), 8.06–8.08 (*d*, Ar–**H**, 1H), 7.99–8.00 (*d*, Ar–**H**, 1H), 7.83 (*s*, Ar–**H**, 1H), 7.70–7.74 (*m*, Ar–**H**, 1H), 7.58–7.61 (*m*, Ar–**H**, 1H), 7.48–7.52 (*m*, Ar–**H**, 2H), 7.43–7.46 (*m*, Ar–**H**, 2H), 7.02–7.04 (*m*, Ar–**H**, 2H), 4.03–4.05 (*t*, Ar–O–**CH**_**2**_, 2H), 3.68–3.87 (*m*, O–**CH**_**2**_–CH_3_, 6H), 1.80–1.86 (*br*, Ar–OCH_2_–**CH**_**2**_, 2H), 1.21–1.65 (*br*, 23H), 0.61–0.64 (*t*, Si–**CH**_**2**_, 2H). ^13^C NMR (100 MHz, CDCl_3_, Figure S5b), δ (ppm): 10.39, 14.12, 18.30, 22.69, 26.11, 29.10, 29.34, 29.69, 33.20, 58.29, 68.12, 114.34, 121.87, 123.24, 123.38, 124.66, 125.12, 126.89, 127.83, 128.30, 131.03, 131.22, 132.56, 136.67, 138.29, 139.75.

### Synthesis of Perylen-3-ylmethanol (Pe–OH)

3-Formylperylene (841 mg, 3 mmol) was dissolved in anhydrous THF/MeOH (60:12 mL), followed by the addition of NaBH_4_ (170 mg, 4.5 mmol). The reaction proceeded for 6 h under room temperature in an environment filled with Ar and quenched by water. EA was used to dilute the crude product, followed by sequential extractions with water and brine. The organic phase was then dried over anhydrous MgSO_4_ and subsequently filtered. The solvent was removed using a rotary evaporator to obtain a yellow liquid of 910 mg (quantitative yield). ^1^H NMR (500 MHz, DMSO-*d*_6_, Figure S6a), δ (ppm): 8.33–8.38 (*m*, Ar–**H**, 4H), 7.91–7.93 (*d*, Ar–**H**, 1H), 7.76–7.78 (*d*, Ar–**H**, 2H), 7.51–7.60 (*m*, Ar–**H**, 4H), 5.34–5.36 (*t*, Ar–CH_2_–O**H**, 2H), 4.90–4.91 (*d*, Ar–**CH**_**2**_–O, 2H). ^13^C NMR (100 MHz, DMSO-*d*_6_, Figure S6b), δ (ppm): 61.15, 120.29, 120.43, 120.59, 120.69, 123.81, 125.14, 126.73, 126.87, 127.68, 127.90, 127.99, 129.52, 130.58, 130.77, 131.81, 134.26, 137.90.

### Synthesis of 3-((Dec-9-en-1-yloxy)methyl)perylene (Pe-C10)

NaH was first washed with hexanes, and then NaH (136 mg, 5.66 mmol) and Pe–OH (800 mg, 2.83 mmol) were dissolved in anhydrous THF (44 mL). The reaction proceeded for 1 h under 45 °C in an environment filled with Ar. After that, TBAB (91.2 mg, 0.28 mmol) was added to the solution. 10-Bromo-1-decene (0.85 mL, 4.25 mmol) was dissolved in anhydrous THF (2 mL) and then added dropwise to the solution. The reaction was conducted at 45 °C for 18 h in an Ar environment and quenched by water. EA was used to dilute the crude product, followed by sequential extractions with water and brine. The organic phase was then dried over anhydrous MgSO_4_ and subsequently filtered. The solvent was removed using a rotary evaporator. The crude product was purified by column chromatography with hexanes and EA. Evaporation was conducted to obtain a yellow solid of 521 mg (44% yield). ^1^H NMR (500 MHz, CDCl_3_, Figure S7a), δ (ppm): 8.12–8.21 (*m*, Ar–**H**, 4H), 7.92–7.93 (*d*, Ar–**H**, 1H), 7.65–7.67 (*d*, Ar–**H**, 2H), 7.44–7.52 (*m*, Ar–**H**, 4H), 5.74–5.82 (*m*, CH_2_–**CH**=CH_2_, 1H), 4.89–4.98 (*m*, CH_2_–CH=**CH**_**2**_, 2H), 4.86 (*s*, Ar–**CH**_**2**_–O, 2H), 3.53–3.56 (*t*, Ar–CH_2_O–**CH**_**2**_, 2H), 1.97–2.02 (*m*, **CH**_**2**_–CH=CH_2_, 2H), 1.60–1.66 (*br*, Ar–CH_2_OCH_2_–**CH**_**2**_, 2H), 1.25–1.36 (*br*, 10H). ^13^C NMR (100 MHz, CDCl_3_, Figure S7b), δ (ppm): 26.19, 28.90, 29.07, 29.37, 29.44, 29.78, 33.78, 70.54, 71.44, 114.08, 119.59, 120.18, 120.23, 120.28, 123.93, 126.54, 126.63, 127.05, 127.80, 128.47, 129.00, 131.15, 131.32, 131.52, 133.00, 133.78, 134.61, 139.22.

### Synthesis of Triethoxy(10-(perylen-3-ylmethoxy)decyl)silane (Pe)

A mixture of Pe-C10 (450 mg, 1.07 mmol), triethoxysilane (0.4 mL, 2.14 mmol), and Karstedt’s catalyst (10.7 μL) was dissolved in anhydrous toluene (10.7 mL), and the reaction was conducted at 70 °C for 16 h in an Ar environment. DCM was used to dilute the crude product, followed by sequential extractions with water and brine. The organic phase was then dried over anhydrous MgSO_4_ and subsequently filtered. The solvent was removed using a rotary evaporator to obtain a yellow liquid of 614 mg (98% yield). ^1^H NMR (500 MHz, CDCl_3_, Figure S8a), δ (ppm): 8.12–8.21 (*m*, Ar–**H**, 4H), 7.92–7.94 (*d*, Ar–**H**, 1H), 7.65–7.67 (*d*, Ar–**H**, 2H), 7.44–7.53 (*m*, Ar–**H**, 4H), 4.86 (*s*, Ar–**CH**_**2**_–O, 2H), 3.77–3.87 (*m*, O–**CH**_**2**_–CH_3_, 6H), 3.53–3.56 (*t*, Ar–CH_2_O–**CH**_**2**_, 2H), 1.60–1.66 (*br*, Ar–CH_2_OCH_2_–**CH**_**2**_, 2H), 1.18–1.41 (*br*, 23H), 0.58–0.64 (*t*, Si–**CH**_**2**_, 2H). ^13^C NMR (100 MHz, CDCl_3_, Figure S8b), δ (ppm): 10.36, 14.10, 17.99, 18.29, 22.71, 26.22, 29.25, 29.49, 29.65, 29.80, 33.19, 58.27, 59.10, 70.61, 71.44, 119.61, 120.19, 120.24, 120.29, 123.94, 126.56, 126.65, 127.04, 127.80, 128.48, 129.00, 131.16, 131.31, 131.53, 133.01, 133.81, 134.62.

### Synthesis of 4-(Benzo[*b*]benzo[4,5]thieno[2,3-*d*]thiophen-2-yl)phenol (BTBT–OH)

2-Bromo[1]benzothieno[3,2-*b*][1]benzothiophene (108 mg, 0.34 mmol) was dissolved in THF (1 mL), and K_2_CO_3_ (281 mg, 2.03 mmol), 4-hydroxyphenylboronic acid (93.3 mg, 0.68 mmol), and water (0.5 mL) were added to the solution. After adding Pd(PPh_3_)_4_ (3.91 mg, 3.4 μmol), the mixture was freeze pumped immediately. Then, the reaction proceeded at 80 °C for 48 h in an Ar environment. THF in the crude product was removed using a rotary evaporator to avoid emulsification. DCM was used to dilute the crude product, followed by sequential extractions with water and brine. The organic phase was then dried over anhydrous MgSO_4_ and subsequently filtered. The crude product was purified by column chromatography with DCM. Evaporation was conducted to give a white solid of 56.5 mg (50% yield). ^1^H NMR (500 MHz, DMSO-*d*_6_, Figure S9a), δ (ppm): 9.63 (*s*, Ar–O**H**, 1H), 8.38 (*s*, Ar–**H**, 1H), 8.16–8.17 (*d*, Ar–**H**, 1H), 8.01–8.10 (*m*, Ar–**H**, 2H), 7.76–7.78 (*d*, Ar–**H**, 1H), 7.65–7.66 (*d*, Ar–**H**, 2H), 7.48–7.56 (*m*, Ar–**H**, 2H), 6.90–6.91 (*d*, Ar–**H**, 2H). ^13^C NMR (100 MHz, DMSO-*d*_6_, Figure S9b), δ (ppm): 115.85, 121.53, 121.58, 121.90, 123.78, 124.48, 125.39, 128.06, 130.20, 132.44, 137.75, 141.52, 142.64, 157.39.

### Synthesis of 2-(4-(Dec-9-en-1-yloxy)phenyl)benzo[*b*]benzo[4,5]thieno[2,3-*d*]thiophene (BTBT-C10)

BTBT–OH (80 mg, 0.24 mmol) and K_2_CO_3_ (50 mg, 0.36 mmol) were dissolved in anhydrous DMF (2.4 mL). The reaction proceeded for 1 h under room temperature in an environment filled with Ar. After that, TBAB and 10-bromo-1-decene (dropwise) were added to the solution. The reaction was conducted at 60 °C for 18 h in an Ar environment. DMF in the crude product was removed using a rotary evaporator to avoid emulsification. DCM was used to dilute the crude product, followed by sequential extractions with water and brine. The organic phase was then dried over anhydrous MgSO_4_ and subsequently filtered. The solvent was removed using a rotary evaporator. The crude product was purified by column chromatography with hexanes and DCM. Evaporation was conducted to obtain a white solid of 51 mg (45% yield). ^1^H NMR (500 MHz, CDCl_3_, Figure S10a), δ (ppm): 8.06 (*s*, Ar–**H**, 1H), 7.86–7.92 (*m*, Ar–**H**, 3H), 7.63–7.65 (*d*, Ar–**H**, 1H), 7.58–7.60 (*d*, Ar–**H**, 2H), 7.37–7.46 (*m*, Ar–**H**, 2H), 6.98–7.00 (*d*, Ar–**H**, 2H), 5.76–5.84 (*m*, CH_2_–**CH**=CH_2_, 1H), 4.91–5.01 (*m*, CH_2_–CH=**CH**_**2**_, 2H), 3.99–4.01 (*t*, Ar–O–**CH**_**2**_, 2H), 2.01–2.06 (*m*, **CH**_**2**_–CH=CH_2_, 2H), 1.77–1.83 (*br*, Ar–OCH_2_–**CH**_**2**_, 2H), 1.23–1.39 (*br*, 10H). ^13^C NMR (100 MHz, CDCl_3_, Figure S10b), δ (ppm): 26.04, 28.91, 29.06, 29.34, 29.41, 29.69, 33.79, 68.13, 114.94, 121.52, 121.70, 121.74, 124.03, 124.16, 124.89, 128.30, 131.64, 133.26, 138.14, 139.19, 142.25, 143.11, 158.92.

### Synthesis of (10-(4-(Benzo[*b*]benzo[4,5]thieno[2,3-*d*]thiophen-2-yl)phenoxy)decyl)triethoxysilane (BTBT)

A mixture of BTBT-C10 (64.4 mg, 0.137 mmol), triethoxysilane (51 μL, 0.274 mmol), and Karstedt’s catalyst (1.37 μL) was dissolved in anhydrous toluene (1.37 mL), and the reaction was conducted at 70 °C for 16 h in an Ar environment. DCM was used to dilute the crude product, followed by sequential extractions with water and brine. The organic phase was then dried over anhydrous MgSO_4_ and subsequently filtered. The solvent was removed using a rotary evaporator to obtain a white solid of 93 mg (quantitative yield). ^1^H NMR (500 MHz, CDCl_3_, Figure S11a), δ (ppm): 8.06 (*s*, Ar–**H**, 1H), 7.86–7.92 (*m*, Ar–**H**, 3H), 7.63–7.65 (*d*, Ar–**H**, 1H), 7.58–7.60 (*d*, Ar–**H**, 2H), 7.37–7.46 (*m*, Ar–**H**, 2H), 6.98–7.00 (*d*, Ar–**H**, 2H), 3.99–4.01 (*t*, Ar–O–**CH**_**2**_, 2H), 3.78–3.85 (*m*, O–**CH**_**2**_–CH_3_, 6H), 1.77–1.83 (m, Ar–CH_2_OCH_2_–**CH**_**2**_, 2H), 1.19–1.26 (*m*, 23H), 0.60–0.64 (*t*, Si–**CH**_**2**_, 2H). ^13^C NMR (100 MHz, CDCl_3_, Figure S11b), δ (ppm): 14.11, 18.30, 22.69, 29.36, 29.70, 31.92, 58.29, 68.15, 114.95, 121.52, 121.71, 121.75, 124.03, 124.17, 124.90, 128.30, 131.64, 133.18, 138.16, 142.25, 143.11, 158.93.

### Characterization

^1^H and ^13^C NMR spectra were analyzed using a Bruker AVIII 500 MHz FT-NMR, and the samples were dissolved in deuterated chloroform (CDCl_3_) or dimethyl sulfoxide (DMSO-*d*_6_). The HOMO levels of the materials were estimated by CV using a CHI 6273E electrochemical analyzer, and the bandgaps (*E*_g_s) were derived from the absorption onset of UV–vis absorption. Then the LUMO levels were calculated as LUMO = HOMO + *E*_g_. TGA (TGA 55, TA Instruments) was measured with 5–20 mg samples at a ramping rate of 10 °C/min from 100 to 700 °C under nitrogen flow rate of 100 mL/min. DSC analysis was conducted using a Discovery DSC 25 from TA Instruments with 3–5 mg samples at a ramping rate of 10 °C/min from −50 to 200 °C. The UV–vis absorption spectra were measured by a Hitachi U-4100 spectrophotometer. PL spectra were obtained using a Jobin Yvon Fluorolog-3 spectrofluorometer with an excitation wavelength of 365 nm. TR-PL with an excitation wavelength of 375 nm was observed using an optical fiber connected to a spectrometer (iHR 320, Horiba) in NSRRC, Taiwan. Given SAMs’ ultrathin thickness (<3 nm), SAM solutions with a concentration of 3 mM were spin-coated at 1000 rpm for 60 s onto ITO glass/quartz for CV/optical analysis.

### Fabrication of the Photosynaptic Device

The conjugated molecules for SAMs were dissolved in anhydrous toluene with a concentration of 3 mM. The SiO_2_ (100 nm)/Si substrate was dipped into the solution at 70 °C for 6 h under 30–40% humidity after oxygen plasma treatment. The SAM-modified substrate was rinsed with toluene using an ultrasonic cleaner and dried in a vacuum overnight. A 50 nm-thick Ph-BTBT-C10 layer was thermally deposited onto the conjugated SAM layer, and a 70 nm-thick Au layer was deposited at a rate of 0.3 Å/s onto the Ph-BTBT-C10 layer using a shadow mask with a channel length (*L*) and width (*W*) of 50 and 1000 μm, respectively.

### Morphological Characterization

XPS was conducted using Thermo Scientific Nexsa G2, and the hydrophilicities of the conjugated SAM layers were determined by contact angle measurement (CAM110, Creating Nano Technologies, Inc.). The morphology analyses of conjugated SAM monolayers and deposited Ph-BTBT-C10 were observed by AFM (Bruker Innova) operating in tapping mode. POM images of Ph-BTBT-C10 were obtained by an Olympus BX51 optical microscope with a U-POT polarizer. ToF-SIMS (TOF.SIMS 5, IONTOF) analysis was conducted to investigate the device architecture. GIWAXS analyzed the molecular alignments of the Ph-BTBT-C10 deposited on different conjugated SAMs at beamline BL13A1 (TLS) in the National Synchrotron Radiation Research Center (NSRRC), Taiwan. A monochromatic beam with a wavelength of 1.027 Å with an incident angle of 0.12° was used.

### Device Characterizations

The phototransistor memory was measured using a Keithley 4200-SCS semiconductor parameter analyzer in a nitrogen-filled glovebox. The transfer characteristic was analyzed by sweeping the *V*_g_ from 20 to −60 V at *V*_d_ = −50 V. The electrical writing process was conducted by applying *V*_g_ = −60 V for 1 s, and light illumination (365 nm; 5.61 mW/cm^2^) for 30 s at *V*_d_ = −50 V with a reading voltage of *V*_g_ = 0 V was conducted for the photoerasing process in the transient characteristic. The synaptic features were measured by a Keithley 2634B in a nitrogen-filled glovebox. The light sources (Titan Electro-Optics Co., Ltd.) were UVA (365 nm; 0.14–5.61 mW/cm^2^), UVB (310 nm; 0.14–0.45 mW/cm^2^), and UVC (265 nm; 0.14–0.74 mW/cm^2^), and the illumination time varied from 50 to 200 ms. The energy consumption was measured at *V*_d_ = −0.1 mV, and other synaptic characteristics were conducted under *V*_d_ = −3 V with a reading voltage of *V*_g_ = 0 V. The light intensities were calibrated by a laser power meter (Thorlabs PM 100D), and all measurements were conducted in a dark environment to prevent interference from external light sources.

### Simulation of Image Denoising and Recognition

The handwritten digital images were generated from the MNIST database. All the simulations were carried out using MATLAB. Random noise with a maximum noise value of 100% was added to the data set, and the images’ brightness value was normalized to 0–255. The denoising array (28 × 28 pixels) was composed of PPF ratio values based on a BNT device with spike interval = 50 ms and 365 nm light intensity of 5.61 mW/cm^2^. The noisy images were repeatedly processed through the denoising array one, three, and five times, and the denoised arrays were converted to gray values to obtain the preprocessed images. An NN size of 784 × 256 × 128 × 10 was constructed for image recognition. 80% of the data set was used for training, and the remaining 20% was for testing.

## Supporting Information Available

^1^H/^13^C NMR, TGA, XPS, and water contact angles of self-assembled molecules; optical, electrochemical, crystallographic, and photophysical parameters of self-assembled molecules and Ph-BTBT-C10; AFM, ToF-SIMS, and GIWAXS analyses of self-assembled molecules and Ph-BTBT-C10; electrical characteristics of the photosynaptic transistors (PDF)
