# Supplementary material for: Revealing the Effect of Crystalline Self-Assembled Monolayer in Biomimetic Photosynapse with Ultraviolet Light Protection Capability
Source: ACS Appl Mater Interfaces. 2024 Dec 5;16(50):69645–59. doi: 10.1021/acsami.4c14221 (PMC11660150; doi:10.1021/acsami.4c14221)
Supplement: Supplementary file 1 — am4c14221_si_001.pdf [file am4c14221_si_001.pdf]

Supporting Information

Revealing the Effect of Crystalline Self-Assembled Monolayer in Biomimetic Photosynapse with Ultraviolet Light Protection Capability

Ya-Shuan Wu,[a] *Wei-Cheng Chen,*[a] *Yi-Sa Lin,*[b] *Cheng-Liang Liu,*[c,d]
Yan-Cheng Lin,[d,e]* *and Wen-Chang Chen*[a,b,d]*

[a] Department of Chemical Engineering, National Taiwan University, Taipei 10617, Taiwan.
[b] Institute of Polymer Science and Engineering, National Taiwan University, Taipei 10607, Taiwan.
[c] Department of Materials Science and Engineering, National Taiwan University, Taipei 10617, Taiwan.
[d] Advanced Research Center of Green Materials Science and Technology, National Taiwan University, Taipei 10617, Taiwan.
[e] Department of Chemical Engineering, National Cheng Kung University, Tainan 70101, Taiwan.

*Corresponding author. E-mail: ycl@gs.ncku.edu.tw (Y.-C. Lin); chenwc@ntu.edu.tw (W.-C. Chen)

Synthesis of 1-((dec-9-en-1-yloxy)methyl)pyrene (Py-C10)

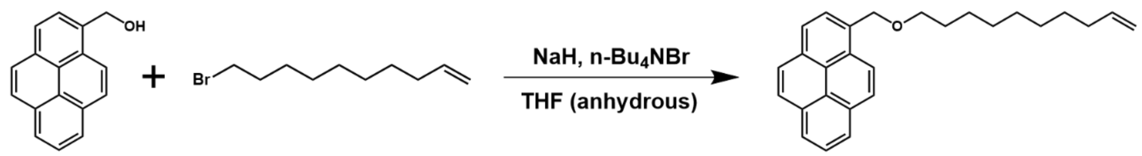

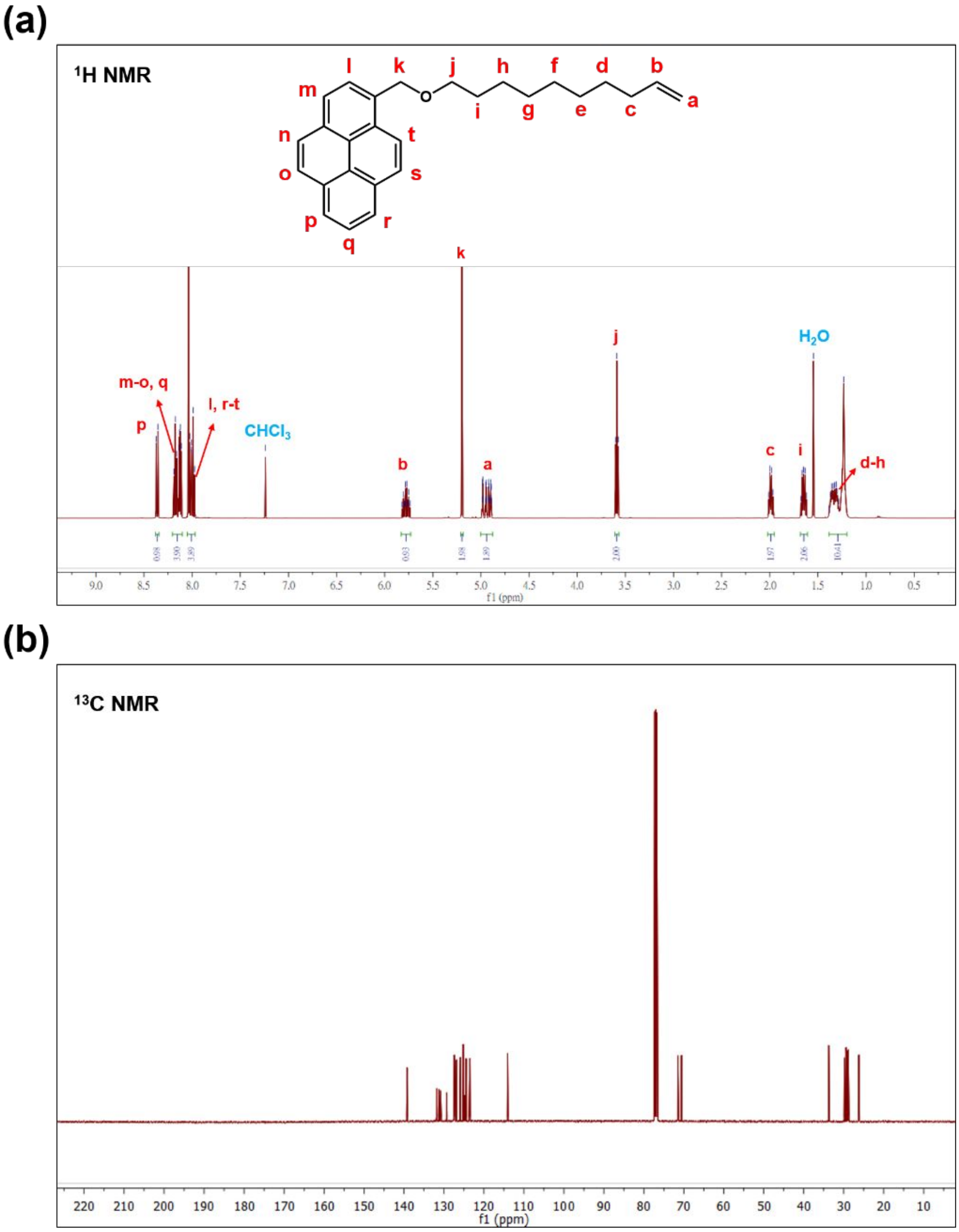

Figure S1. (a) ^{1}H NMR (b) ^{13}C NMR spectra of Py-C10 in $CDCl_3$.

Synthesis of triethoxy(10-(pyren-1-ylmethoxy)decyl)silane (Py)

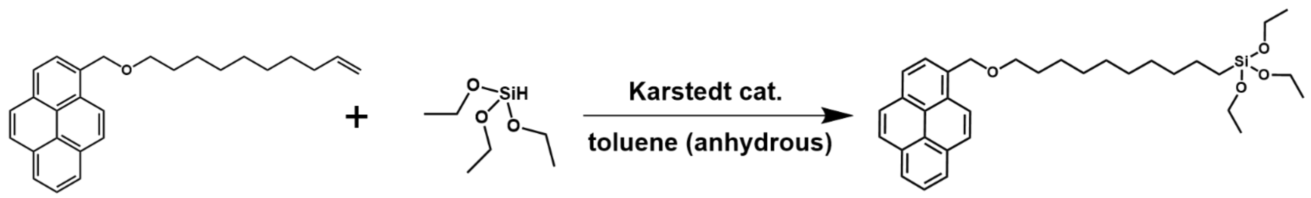

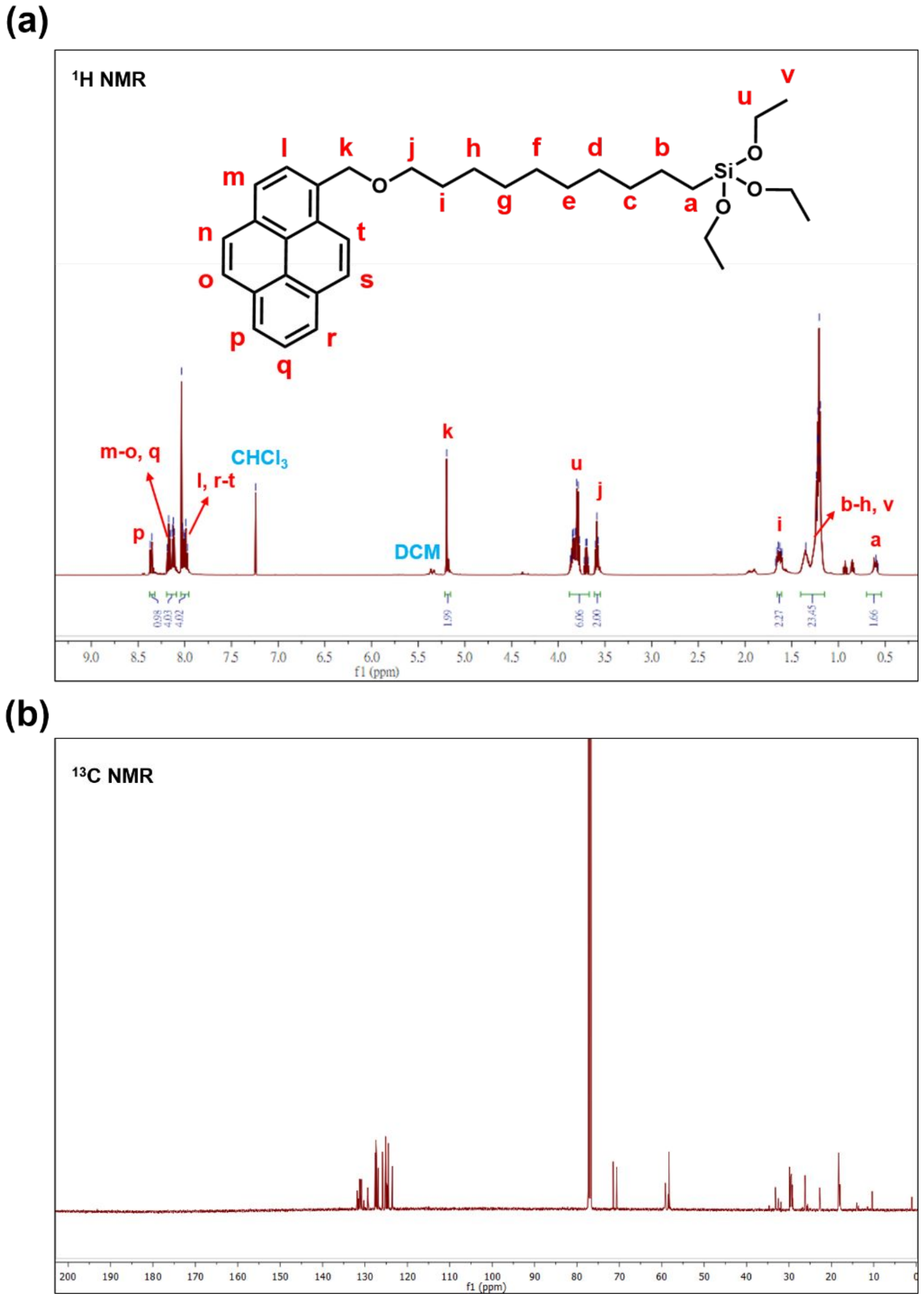

Figure S2. (a) ^{1}H NMR (b) ^{13}C NMR spectra of Py in $CDCl_3$.

Synthesis of 4-(benzo[*b*]naphtho[1,2-*d*]thiophen-5-yl)phenol (BNT-OH)

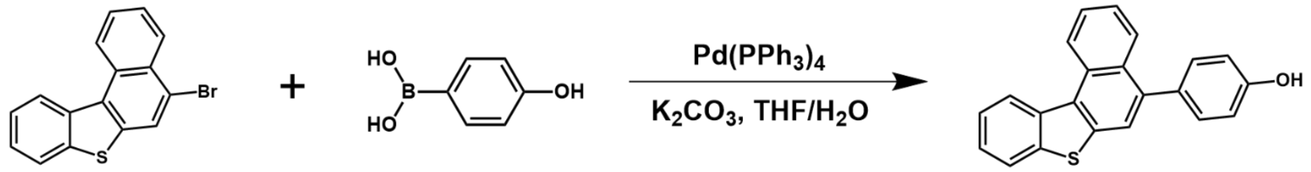

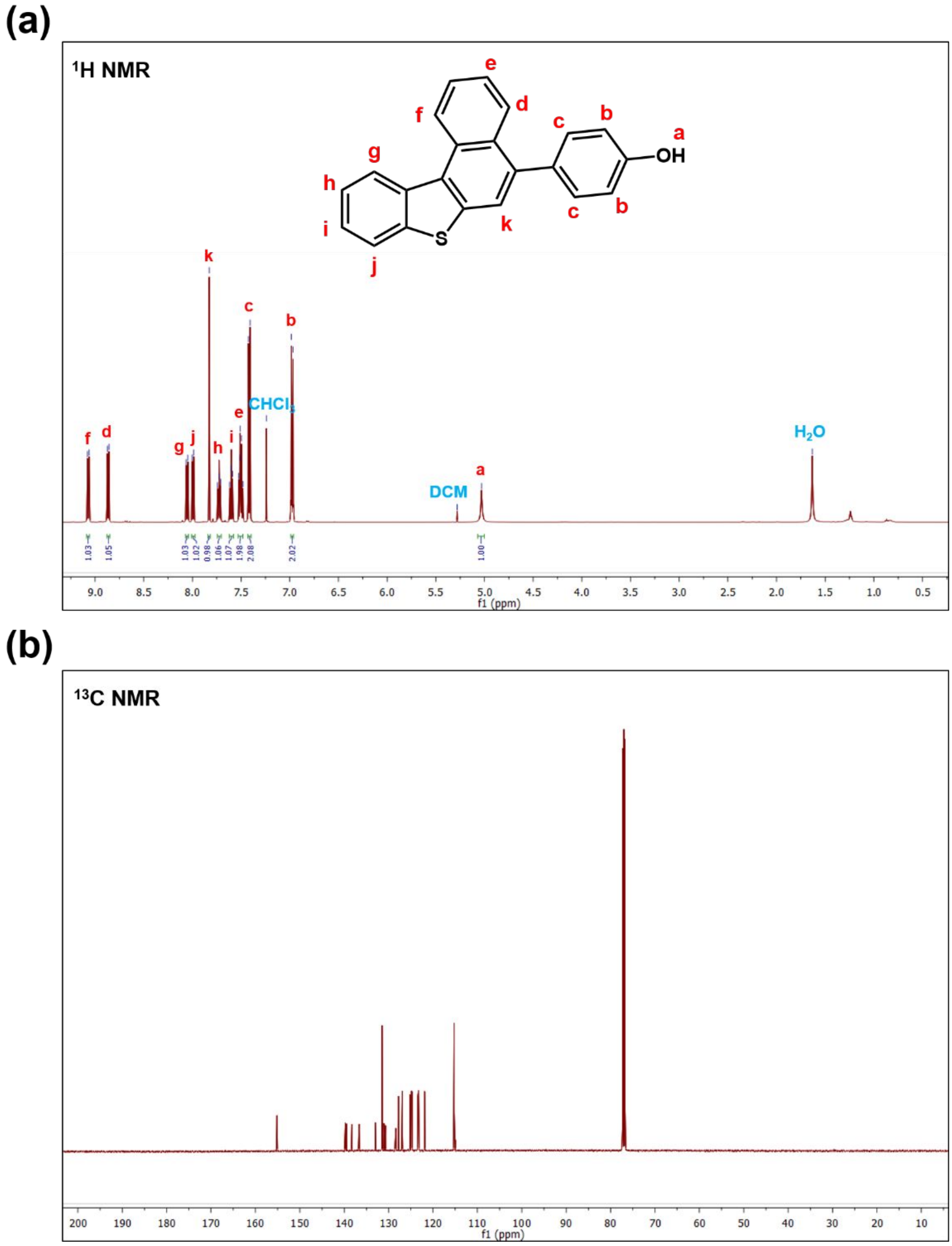

Figure S3. (a) ^{1}H NMR (b) ^{13}C NMR spectra of BNT-OH in $CDCl_3$.

Synthesis of 5-(4-(dec-9-en-1-yloxy)phenyl)benzo[*b*]naphtho[1,2-*d*]thiophene (BNT-C10)

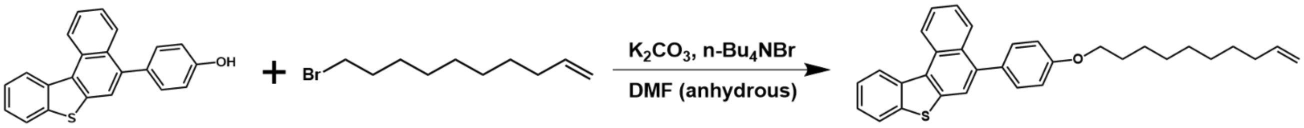

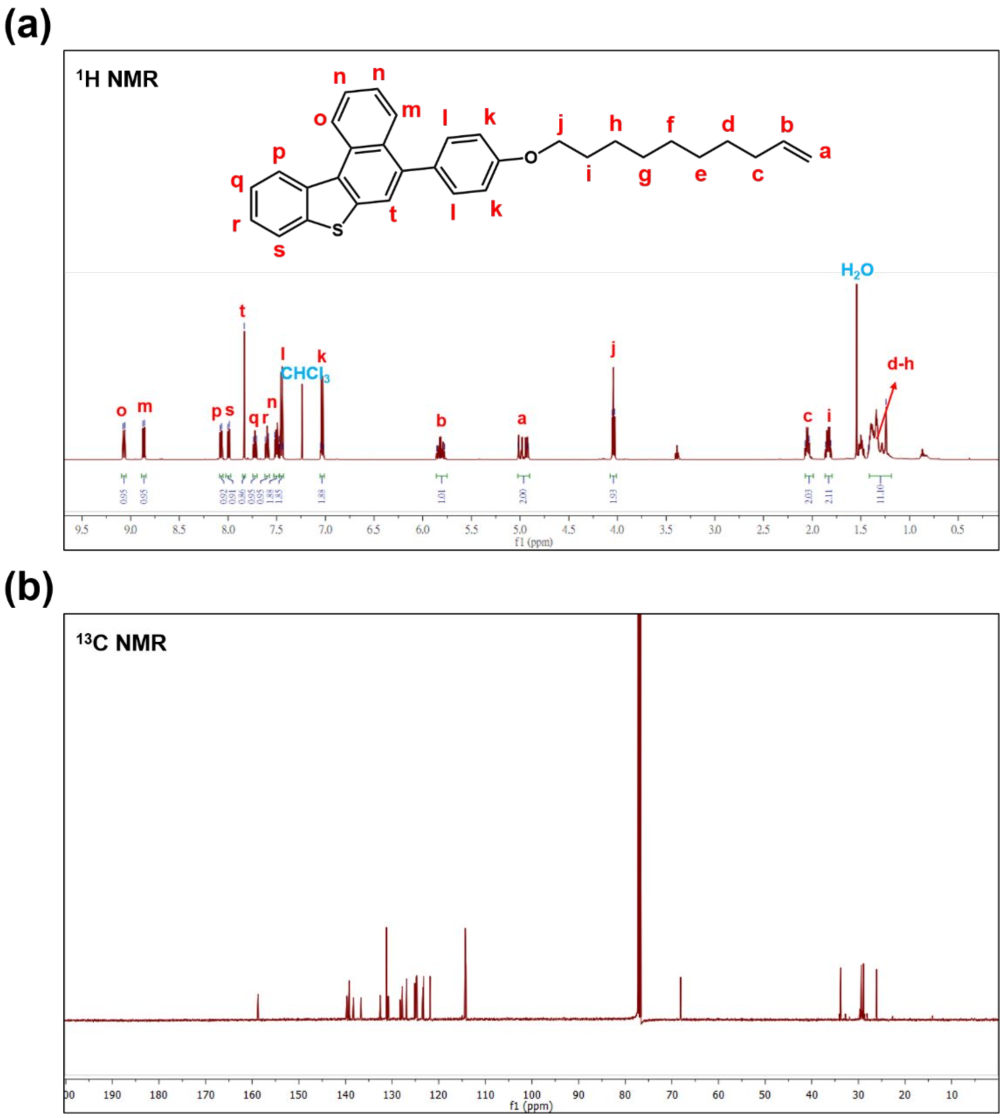

Figure S4. (a) ^{1}H NMR (b) ^{13}C NMR spectra of BNT-C10 in $CDCl_3$.

Synthesis of (10-(4-(benzo[*b*]naphtho[1,2-*d*]thiophen-5-yl)phenoxy)decyl)triethoxysilane (BNT)

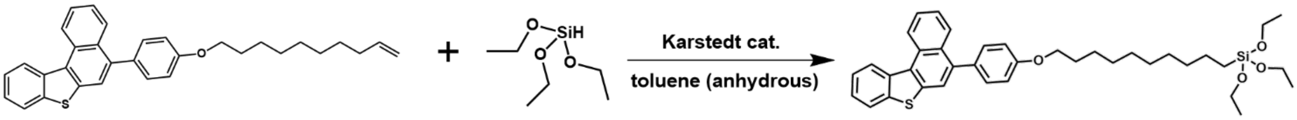

(a)

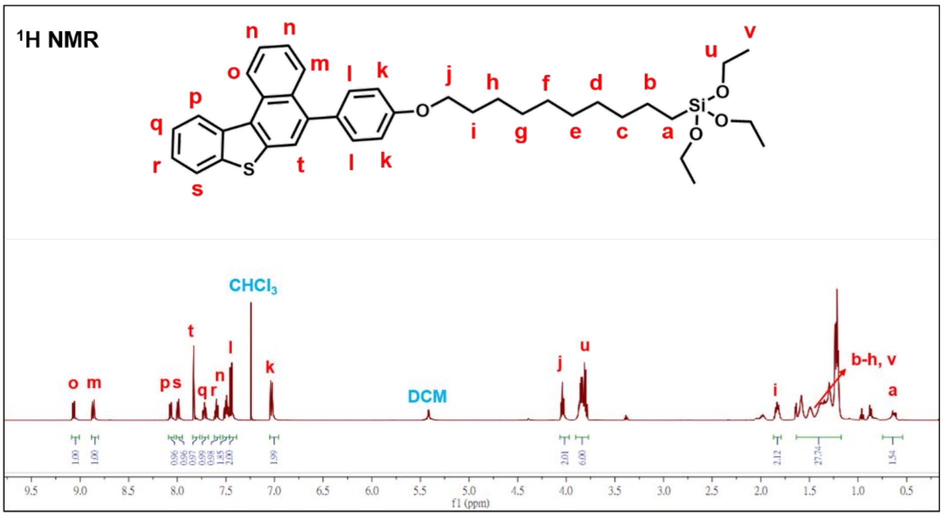

(b)

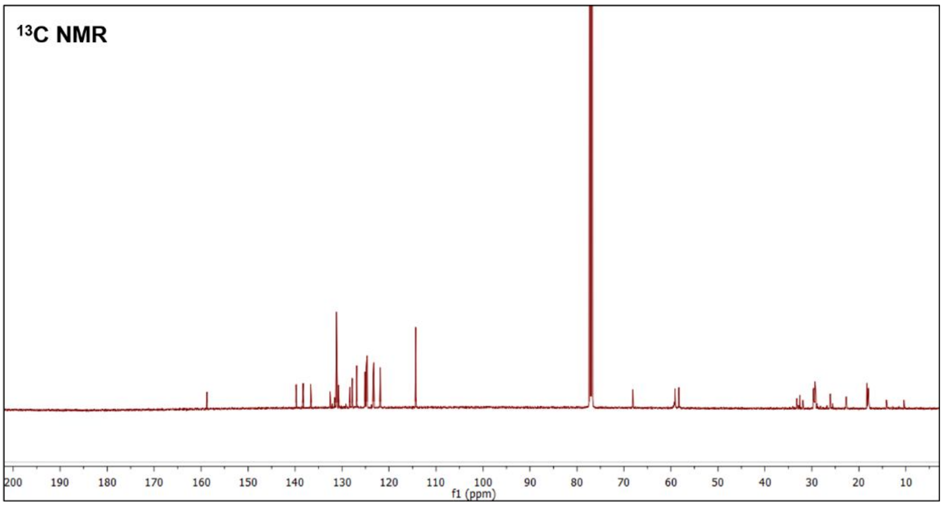

Figure S5. (a) ^{1}H NMR (b) ^{13}C NMR spectra of BNT in $CDCl_3$.

Synthesis of perylen-3-ylmethanol (Pe-OH)

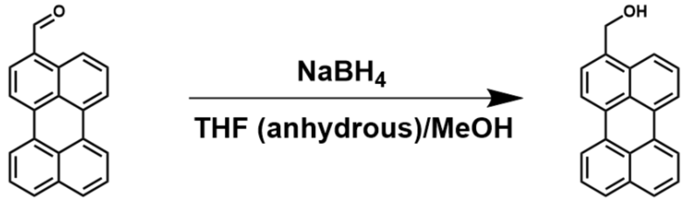

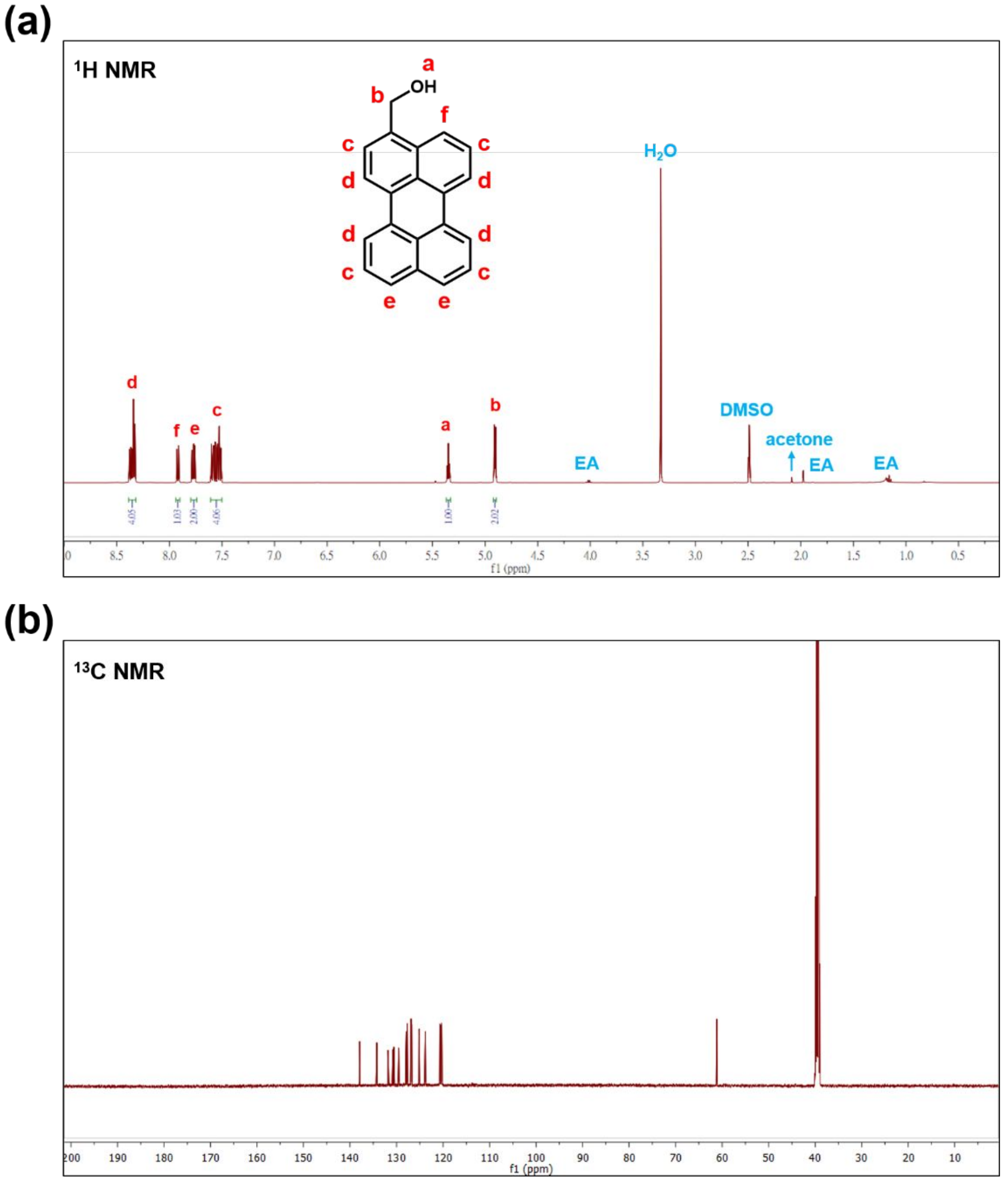

Figure S6. (a) ^{1}H NMR (b) ^{13}C NMR spectra of Pe-OH in DMSO-d_6.

Synthesis of 3-((dec-9-en-1-yloxy)methyl)perylene (Pe-C10)

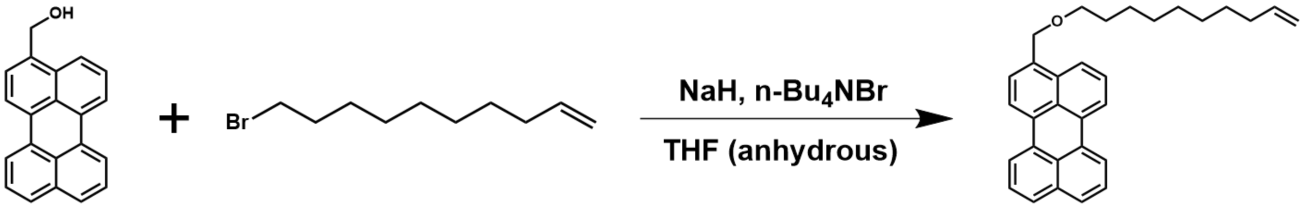

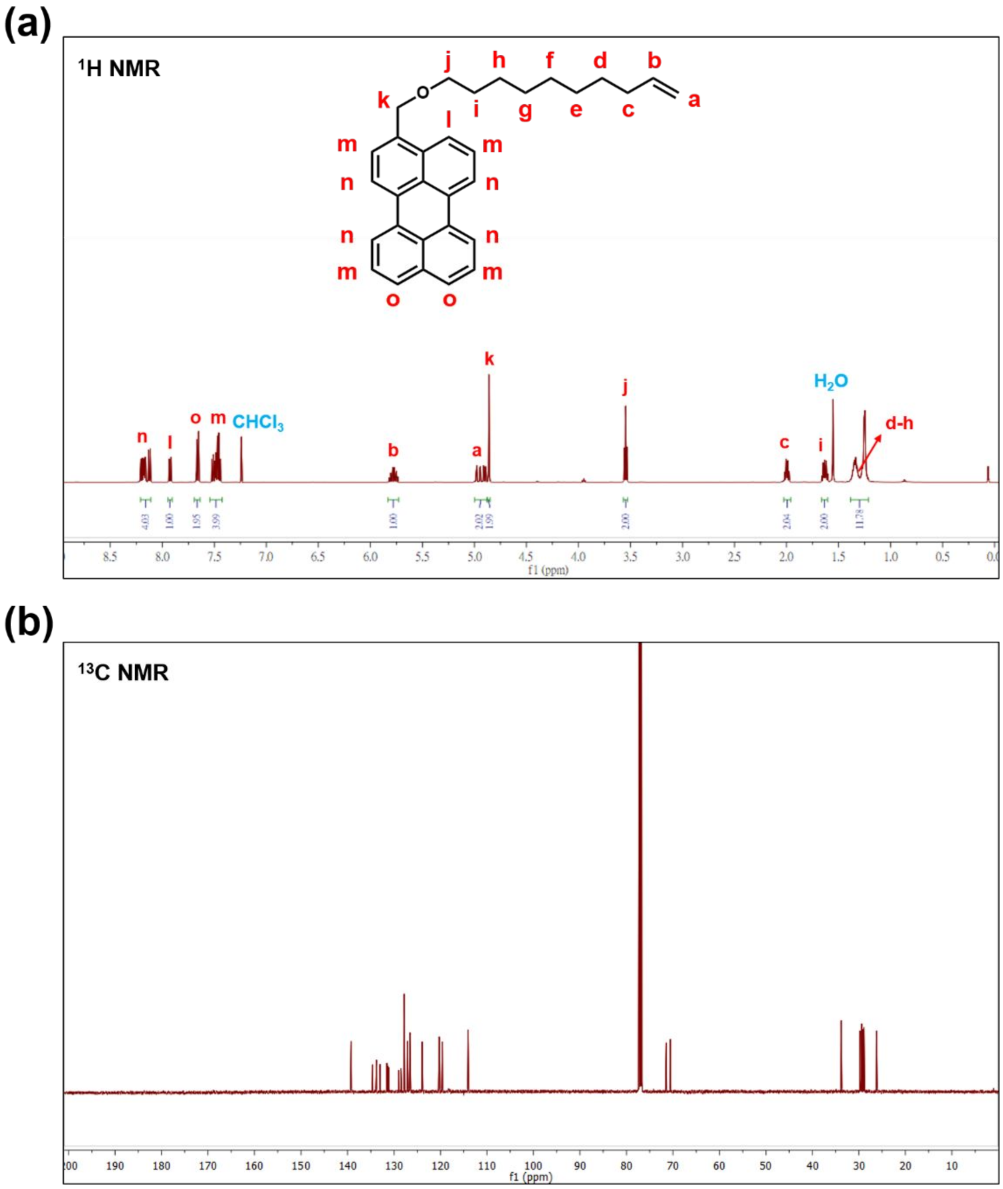

Figure S7. (a) ^{1}H NMR (b) ^{13}C NMR spectra of Pe-C10 in $CDCl_3$.

Synthesis of triethoxy(10-(perylen-3-ylmethoxy)decyl)silane (Pe)

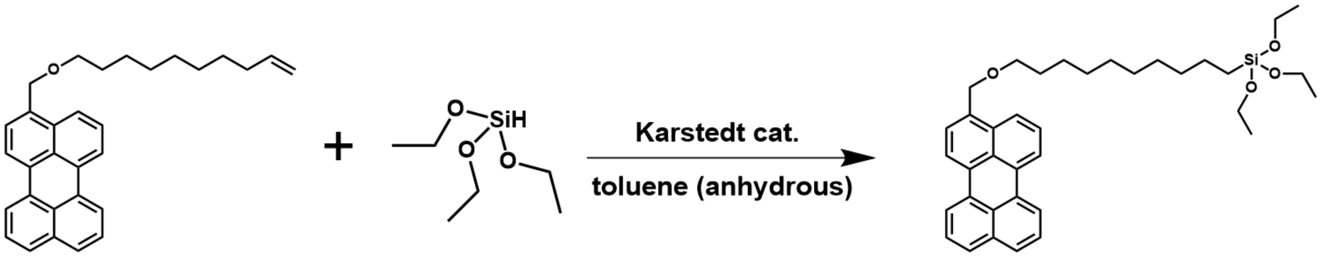

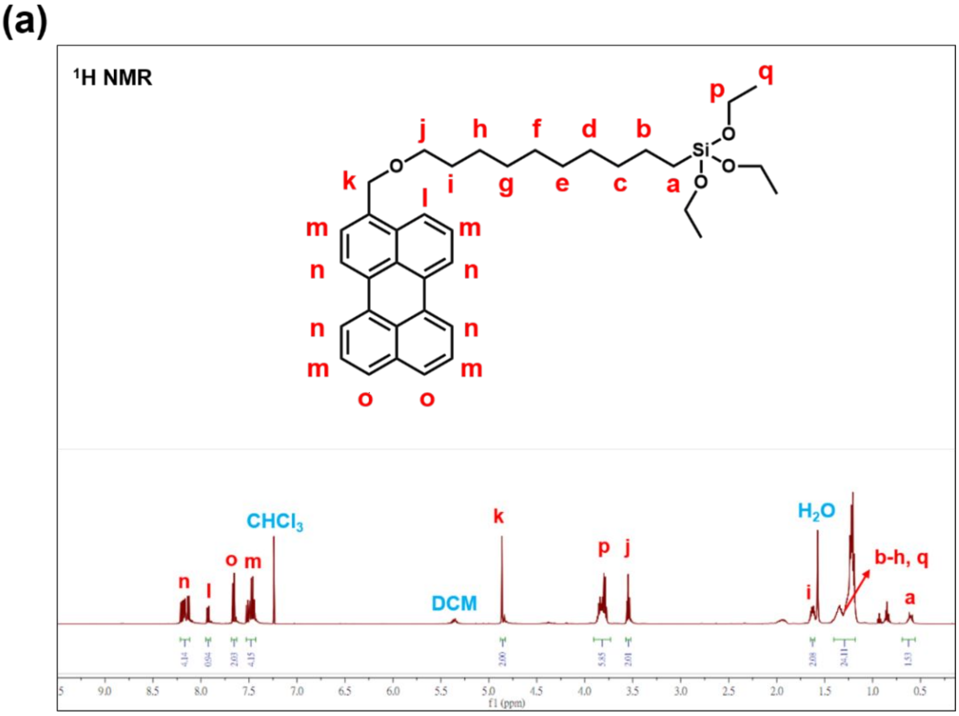

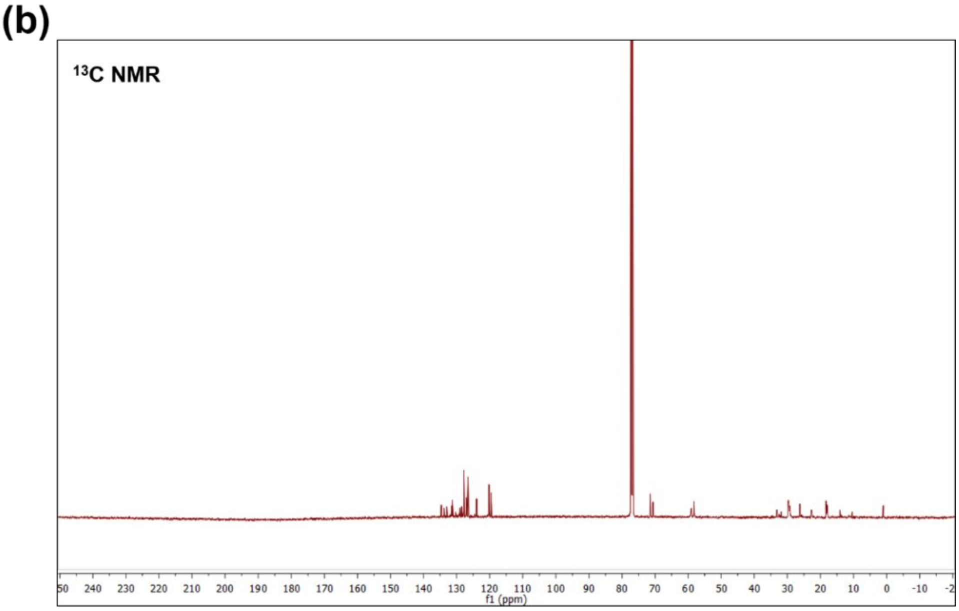

Figure S8. (a) ^{1}H NMR (b) ^{13}C NMR spectra of Pe in $CDCl_3$.

Synthesis of 4-(benzo[*b*]benzo[4,5]thieno[2,3-*d*]thiophen-2-yl)phenol (BTBT-OH)

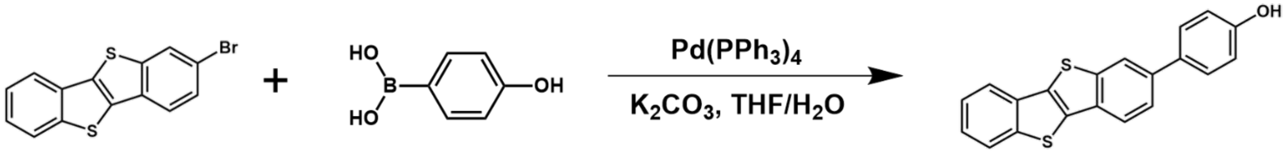

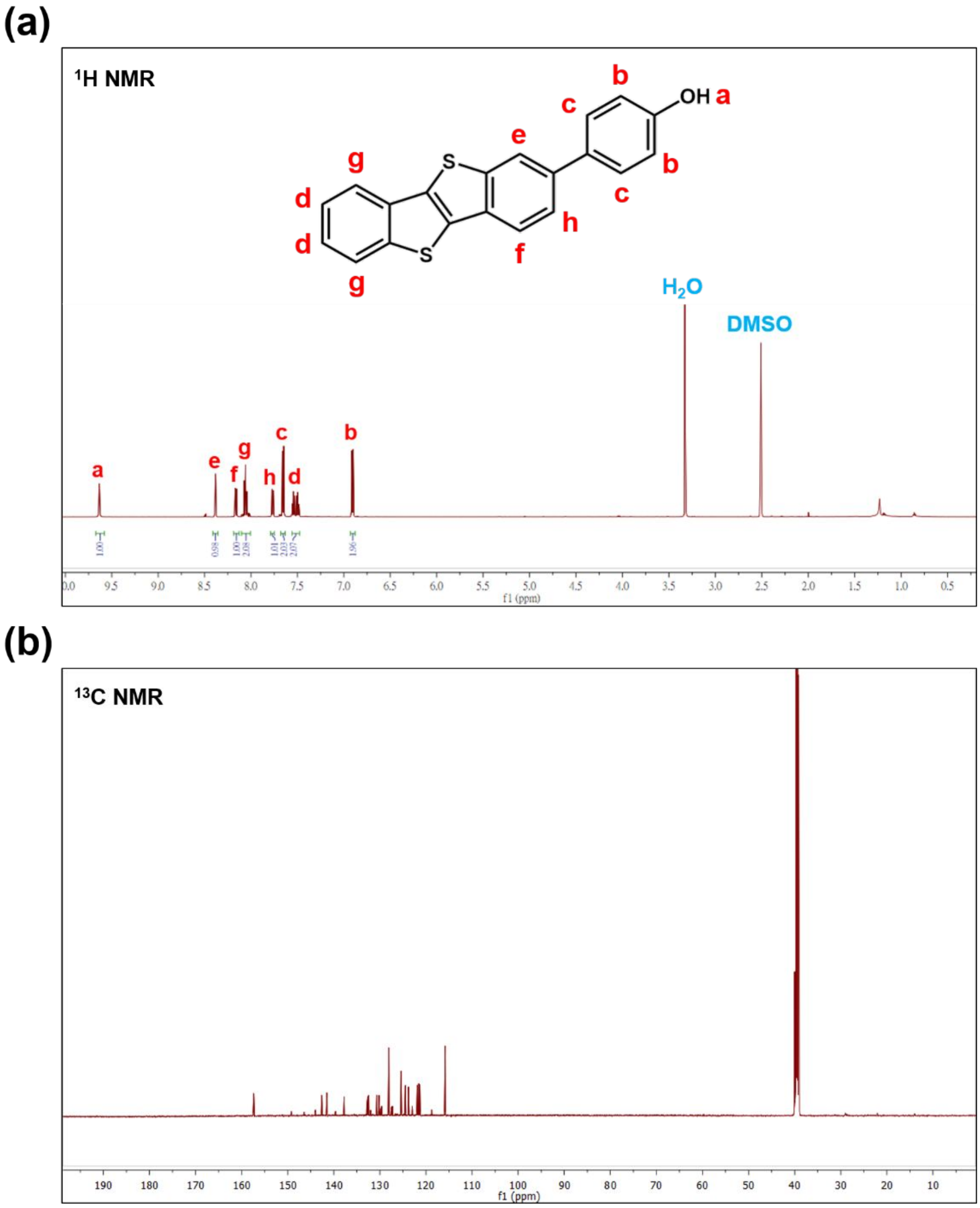

Figure S9. (a) ^{1}H NMR (b) ^{13}C NMR spectra of BTBT-OH in DMSO-d$_6$.

Synthesis of 2-(4-(dec-9-en-1-yloxy)phenyl)benzo[*b*]benzo[4,5]thieno[2,3-*d*]thiophene (BTBT-C10)

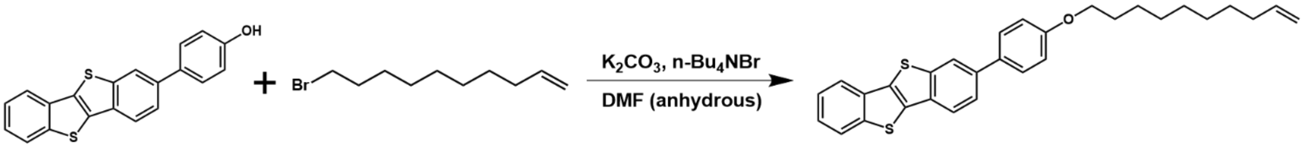

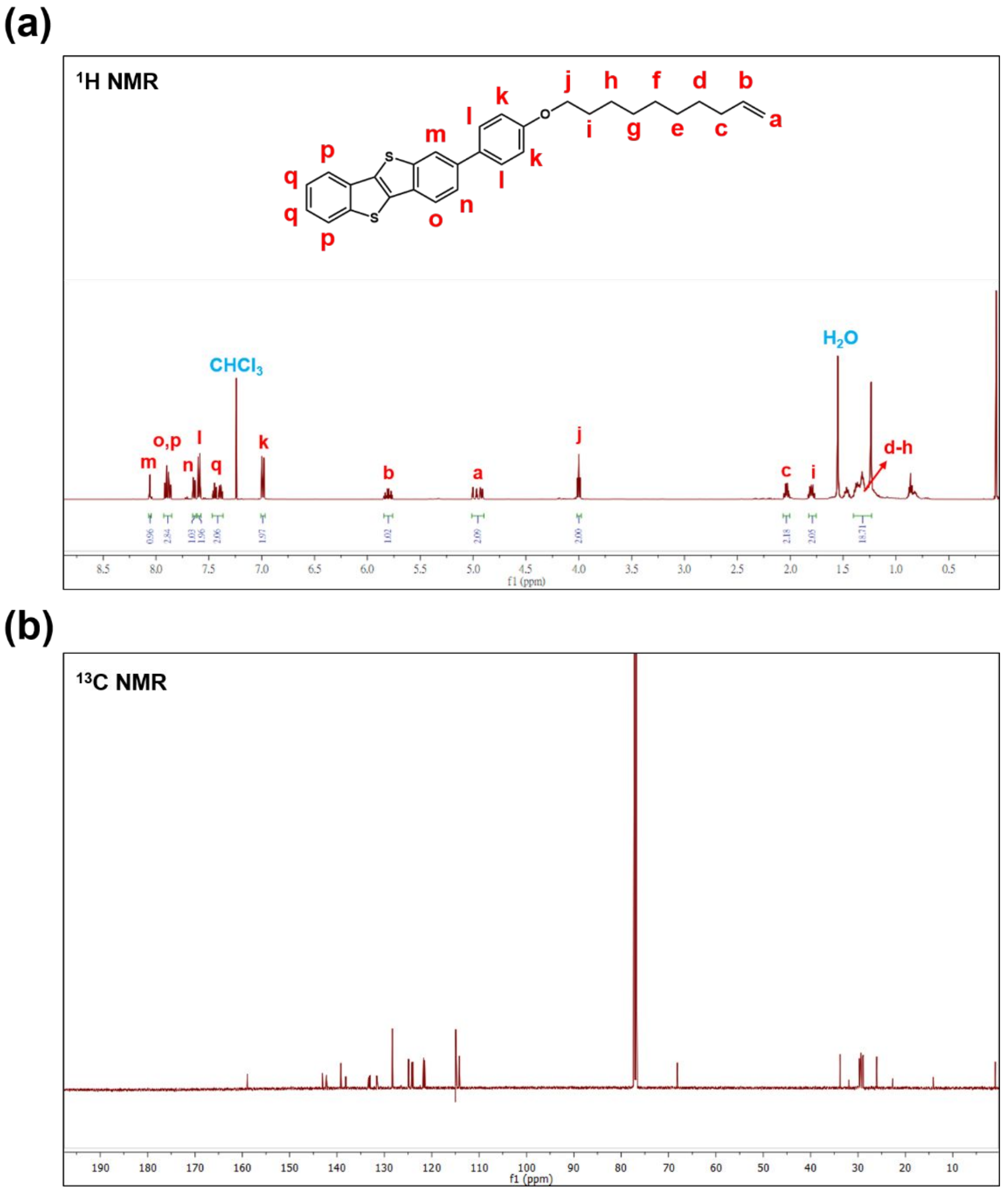

Figure S10. (a) ^{1}H NMR (b) ^{13}C NMR spectra of BTBT-C10 in $CDCl_3$.

Synthesis of (10-(4-(benzo[*b*]benzo[4,5]thieno[2,3-*d*]thiophen-2-yl)phenoxy)decyl)triethoxysilane (BTBT)

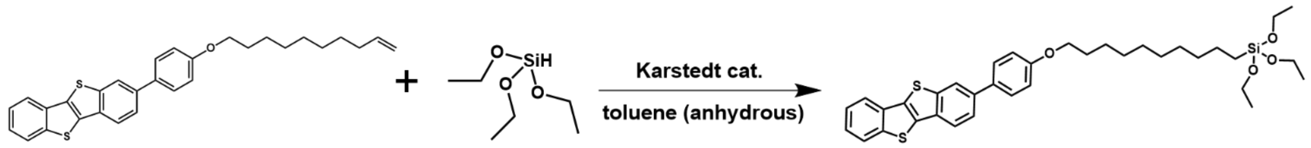

(a)

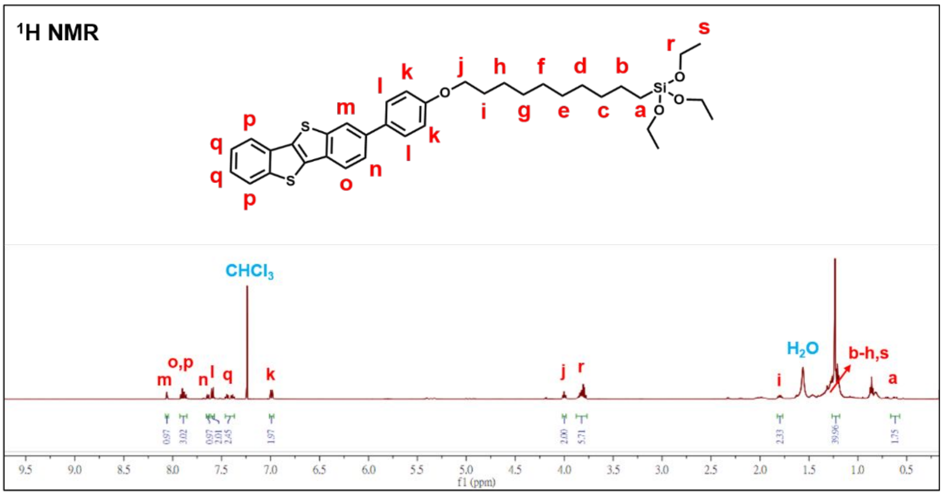

(b)

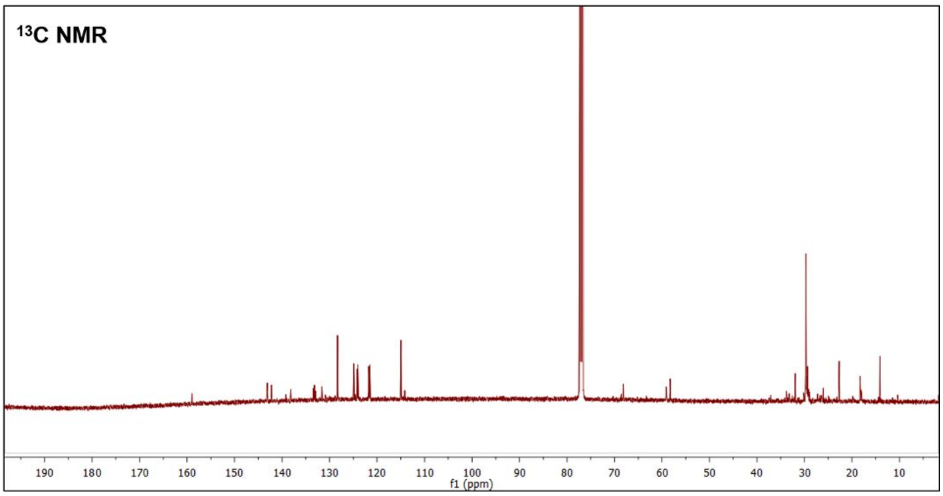

Figure S11. (a) ^{1}H NMR (b) ^{13}C NMR spectra of BTBT in $CDCl_3$.

Table S1. Summary of the SAM-related optoelectronic devices in the literature.

Self-Assembled Material	SAM Thickness	Function of SAM	Operation	Device	Ref.
C3-SAM	–	Surface passivation	Electrical/Optical	Perovskite solar cell	1
4MP, 4ATP, CA, TP	–	Surface passivation	Electrical/Optical	Light emitting diode	2
OTES	–	Surface passivation	Electrical	Transistor	3
C18, DA, ND, DA-ND	20–50 nm	Charge trapping	Electrical	Transistor memory	4
PyPN, PN, DPN	(aggregation)	Charge trapping	Electrical/Optical	Transistor memory	5
Bz, OH, COOH, NH_2	–	Tuning surface energy and passivation	Electrical/Optical	Photodetector	6
CPTS, PhTS, APTS	–	Surface passivation	Electrical/Optical	Photodetector	7
CAAAAKAAAAK, C(GABA)(GABA)D(GABA)(GABA)D, HS-PEG8-CH2CH2COOH	2.5 nm	Conductance modulation	Electrical	Synaptic memristor	8
DPP, IID, BDT, ODTS	~10 nm	Charge trapping	Electrical/Optical	Synaptic transistor	9
CDPA	–	Tuning surface energy	Electrical/Optical	Synaptic transistor	10
Py, BNT, Pe, BTBT	< 3 nm	Charge trapping	Electrical/Optical	Synaptic transistor	This work

Table S2. Summary of the optical and electrochemical properties, including the onset wavelength (λ) in UV–vis absorption spectra and the corresponding bandgap (E_g), the oxidative onset ($E_{ox,onset}$) in CV measurement, and the HOMO/LUMO levels.

	λ (nm)	E_g (eV)	$E_{ox,onset}$ (V)	HOMO (eV)	LUMO (eV)
**Ph-BTBT-C10**	395	3.14	1.44	−5.84	−2.70
**Py**	367	3.38	1.12	−5.52	−2.14
**BNT**	380	3.26	1.36	−5.76	−2.50
**Pe**	497	2.49	0.96	−5.36	−2.87
**BTBT**	396	3.13	1.26	−5.66	−2.53

Table S3. Crystallographic parameters, including domain size, *d*-spacing, paracrystalline disorder, and tilt angle, of the Ph-BTBT-C10 films deposited at different surfaces under room temperature.

R.T.	Domain Size (nm)	d-spacing (nm)	Paracrystalline Disorder (%)	Tilt Angle (°)
**Pristine**	43.90	2.69	9.36	11.27
**Py**	44.95	2.68	9.24	12.28
**BNT**	44.88	2.69	9.26	11.26
**Pe**	40.86	2.60	9.54	18.63
**BTBT**	46.16	2.68	9.13	11.61

Table S4. TR-PL fitting parameters in the average exciton lifetime (τ_{avg}) of Ph-BTBT-C10 films deposited at different surfaces using a single exponential decay function.

	A	τ_{avg} (ns)
**Pristine**	1.04	0.307
**Py**	1.04	0.262
**BNT**	0.95	0.250
**Pe**	1.09	0.269
**BTBT**	1.09	0.269

Table S5. Summary of radiative and nonradiative parameters of Ph-BTBT-C10 films deposited at different surfaces, including PL quenching, photoluminescence quantum yield (PLQY), charge transfer efficiency (CTE), charge transfer rate (k_{CT}), and radiative/nonradiative recombination rates (k_{rad}/k_{nonrad}).

	PL quenching (%)	PLQY (%)	CTE (%)	k_{CT} (ns^{-1})	k_{rad} (ns^{-1})	k_{nonrad} (ns^{-1})
**Pristine**	–	2.54	–	–	0.083	3.17
**Py**	15.1	2.43	14.7	0.56	0.093	3.72
**BNT**	1.6	2.45	18.6	0.74	0.098	3.90
**Pe**	24.8	1.74	12.4	0.46	0.065	3.65
**BTBT**	10.8	2.39	12.4	0.46	0.089	3.63

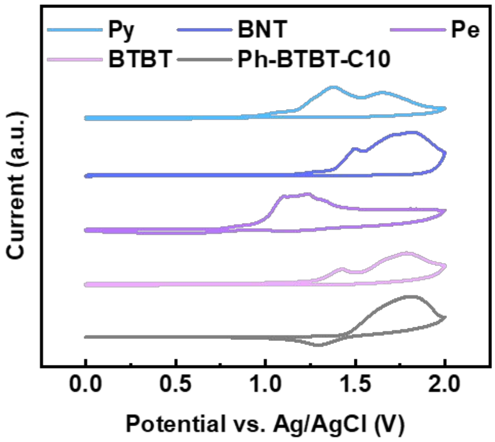

Figure S12. CV profiles of the studied materials investigated using a three-electrode cell comprising an Ag/AgCl reference electrode, a Pt auxiliary electrode, and an ITO glass working electrode coated with the studied materials. The electrolyte solution consisted of 0.1 M tetrabutylammonium perchlorate (TBAP) dissolved in anhydrous acetonitrile, and the cell potential was calibrated using ferrocene.

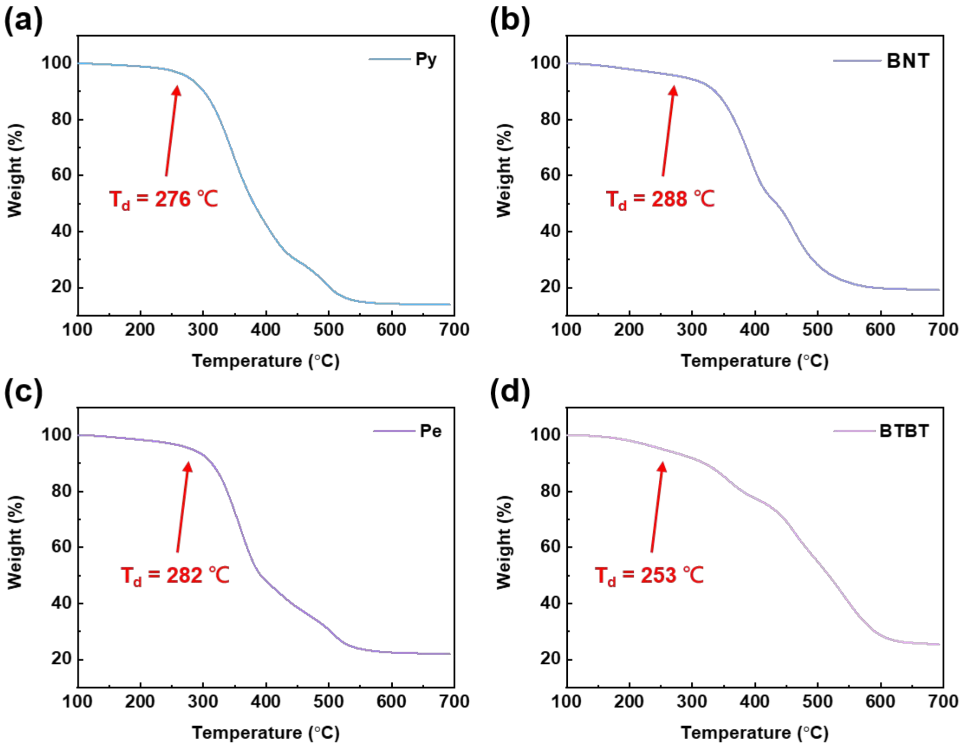

Figure S13. TGA curves of the (a) Py, (b) BNT, (c) Pe, and (d) BTBT under an N_2 atmosphere.

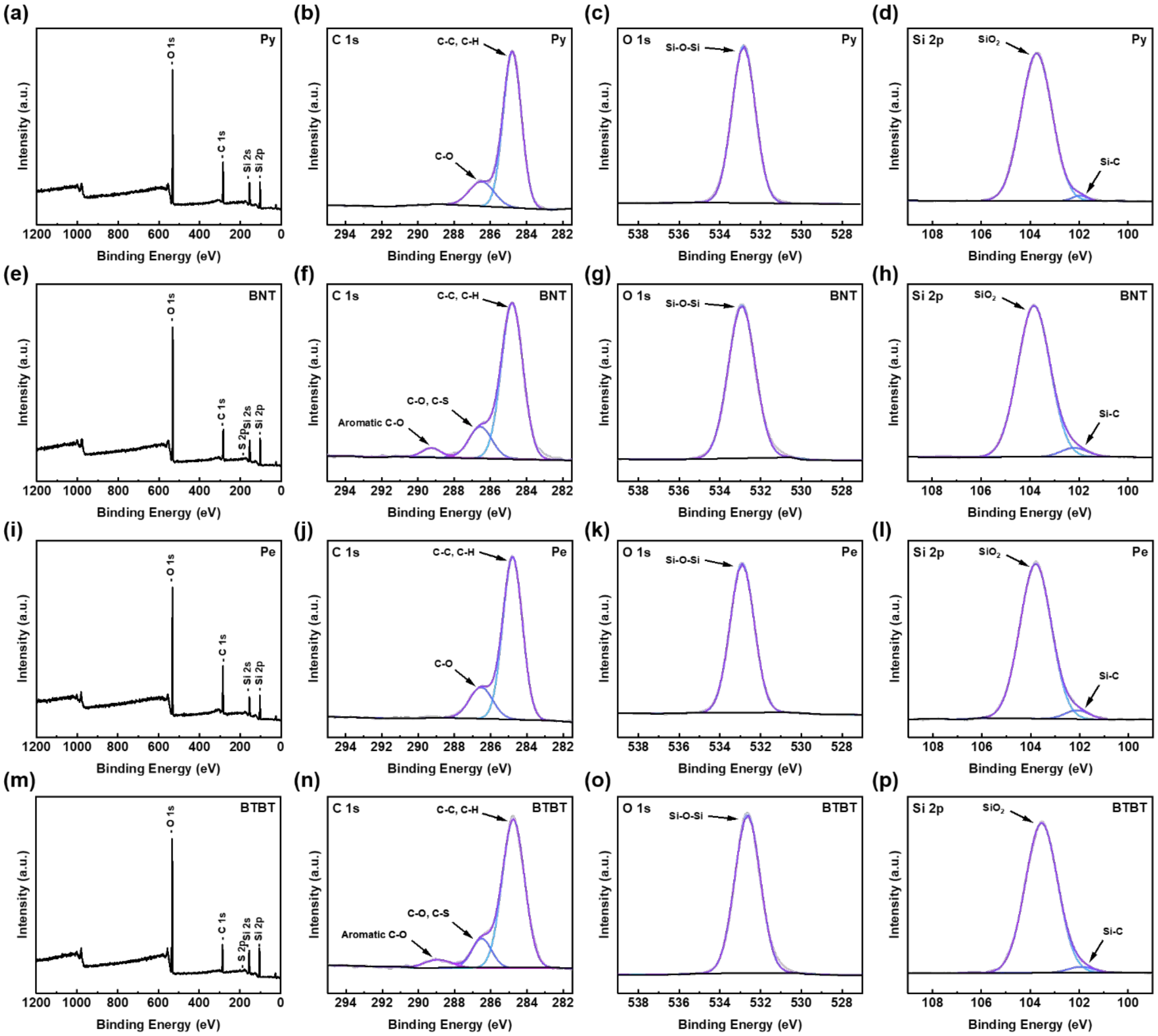

Figure S14. XPS full-spectrum of (a) Py, (e) BNT, (i) Pe, and (m) BTBT. The corresponding peak deconvolutions of (b,f,j,n) C-1s, (c,g,k,o) O-1s, and (d,h,i,p) Si-2p bands for (b–d) Py, (f–h) BNT, (j–l) Pe, and (n–p) BTBT.

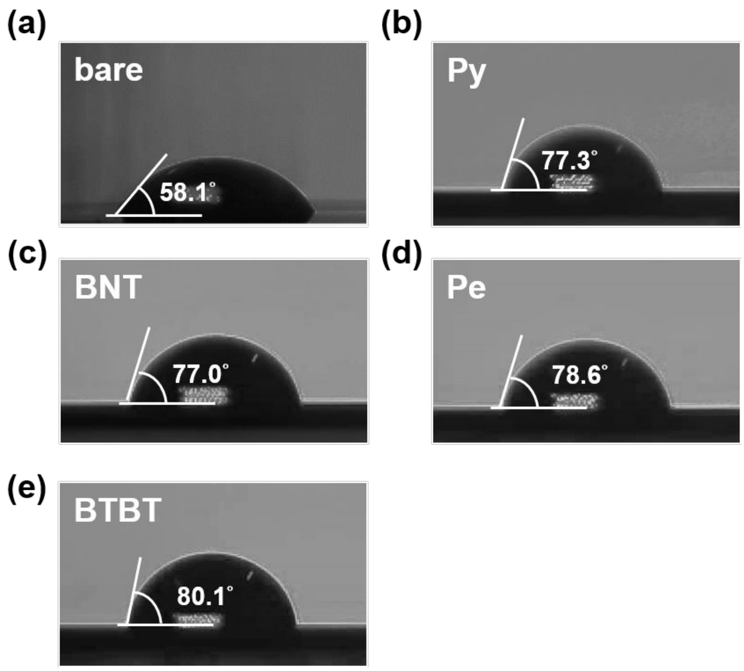

Figure S15. Water contact angles of (a) bare wafer, (b) Py, (c) BNT, (d) Pe, and (e) BTBT SAMs.

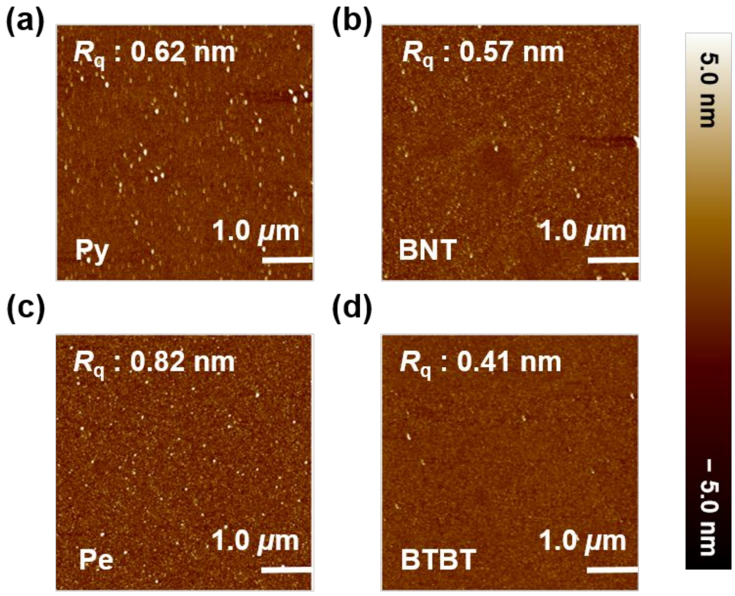

Figure S16. AFM height images of (a) Py, (b) BNT, (c) Pe, and (d) BTBT SAM surfaces before thermal annealing.

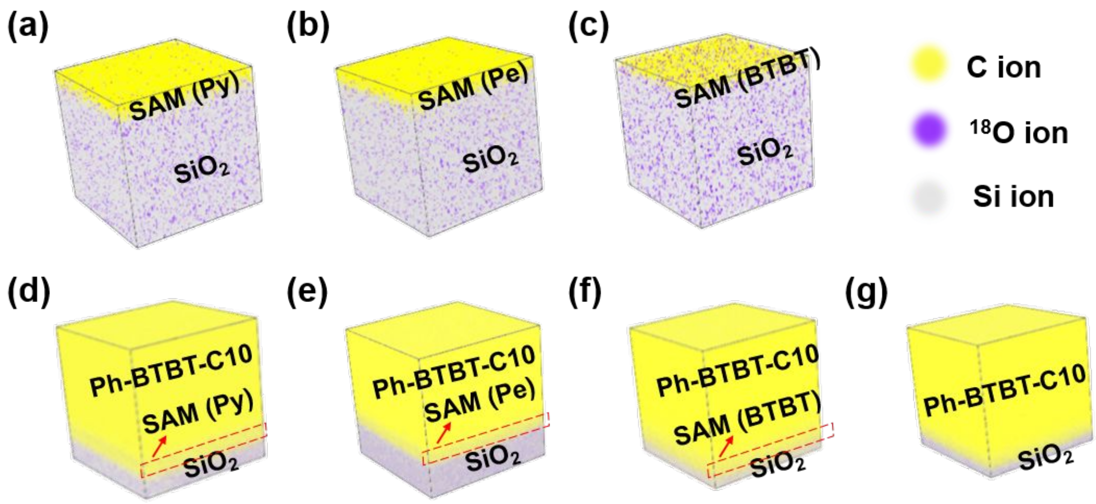

Figure S17. ToF-SIMS 3D mappings of C, ^{18}O, and Si ions for (a) Py/SiO_2, (b) Pe/SiO_2, (c) BTBT/SiO_2, (d) Ph-BTBT-C10/Py/SiO_2, (e) Ph-BTBT-C10/Pe/SiO_2, (f) Ph-BTBT-C10/BTBT/SiO_2, and (g) Ph-BTBT-C10/SiO_2 structures.

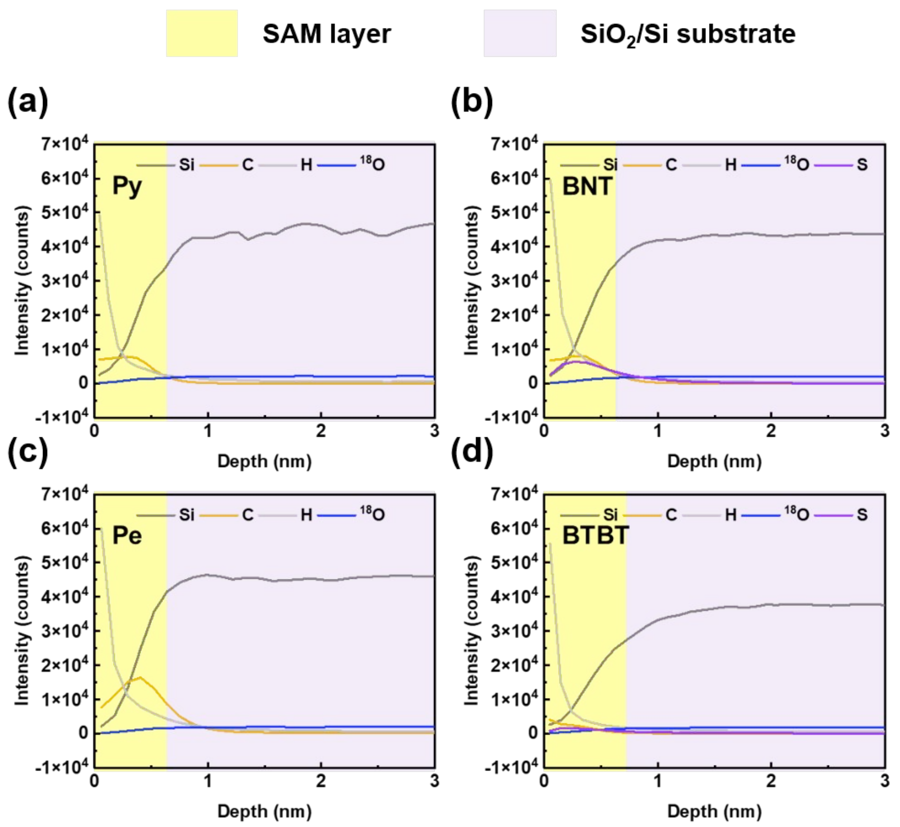

Figure S18. ToF-SIMS 1D profiles of C, H, ^{18}O, and Si ions for (a) Py/SiO_2, (b) BNT/SiO_2, (c) Pe/SiO_2, (d) BTBT/SiO_2 structures.

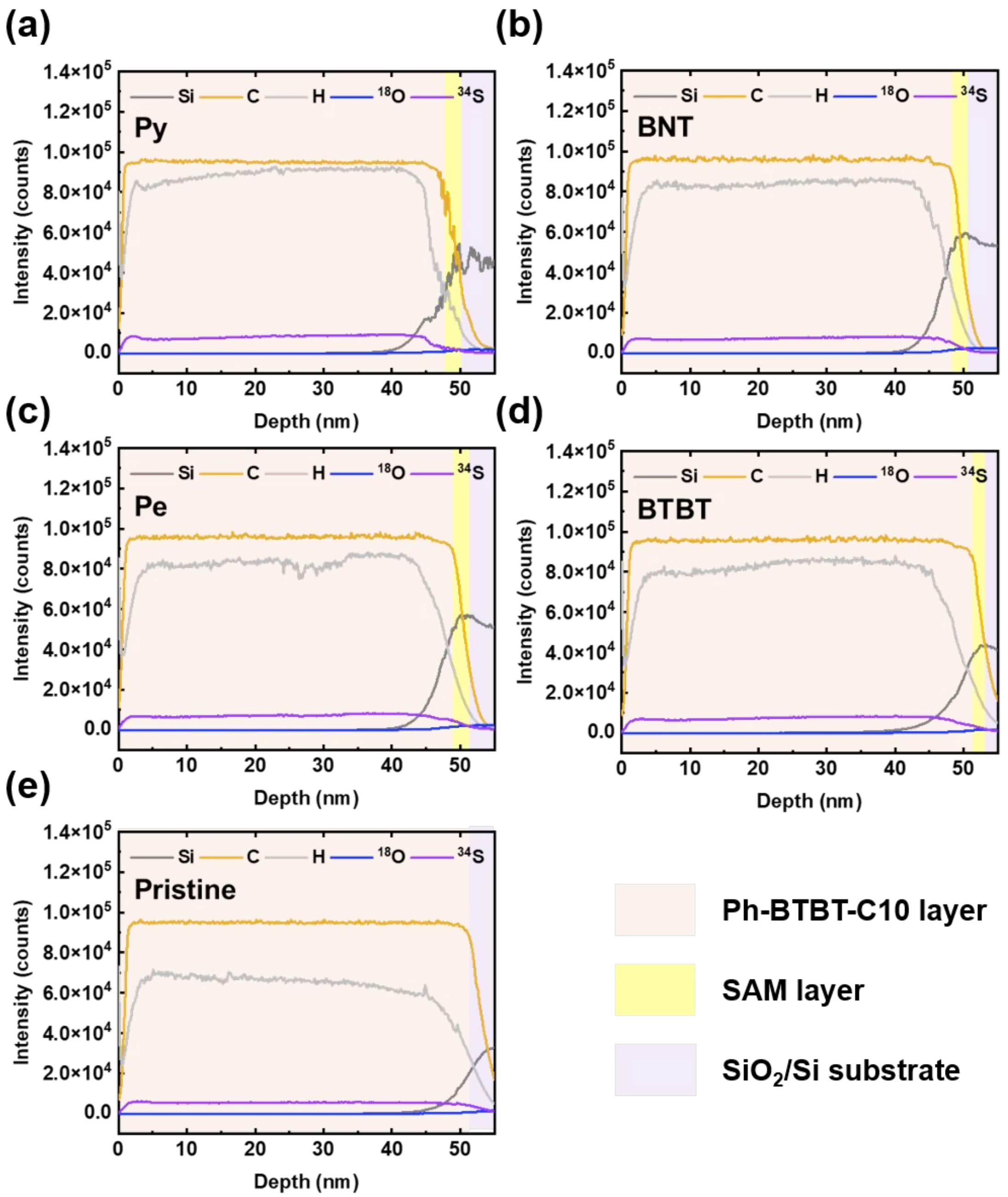

Figure S19. ToF-SIMS 1D profiles of C, H, ^{18}O, and Si ions for (a) Ph-BTBT-C10/Py/SiO_2, (b) Ph-BTBT-C10/BNT/SiO_2, (c) Ph-BTBT-C10/Pe/SiO_2, (d) Ph-BTBT-C10/BTBT/SiO_2, and (e) Ph-BTBT-C10/SiO_2 structures.

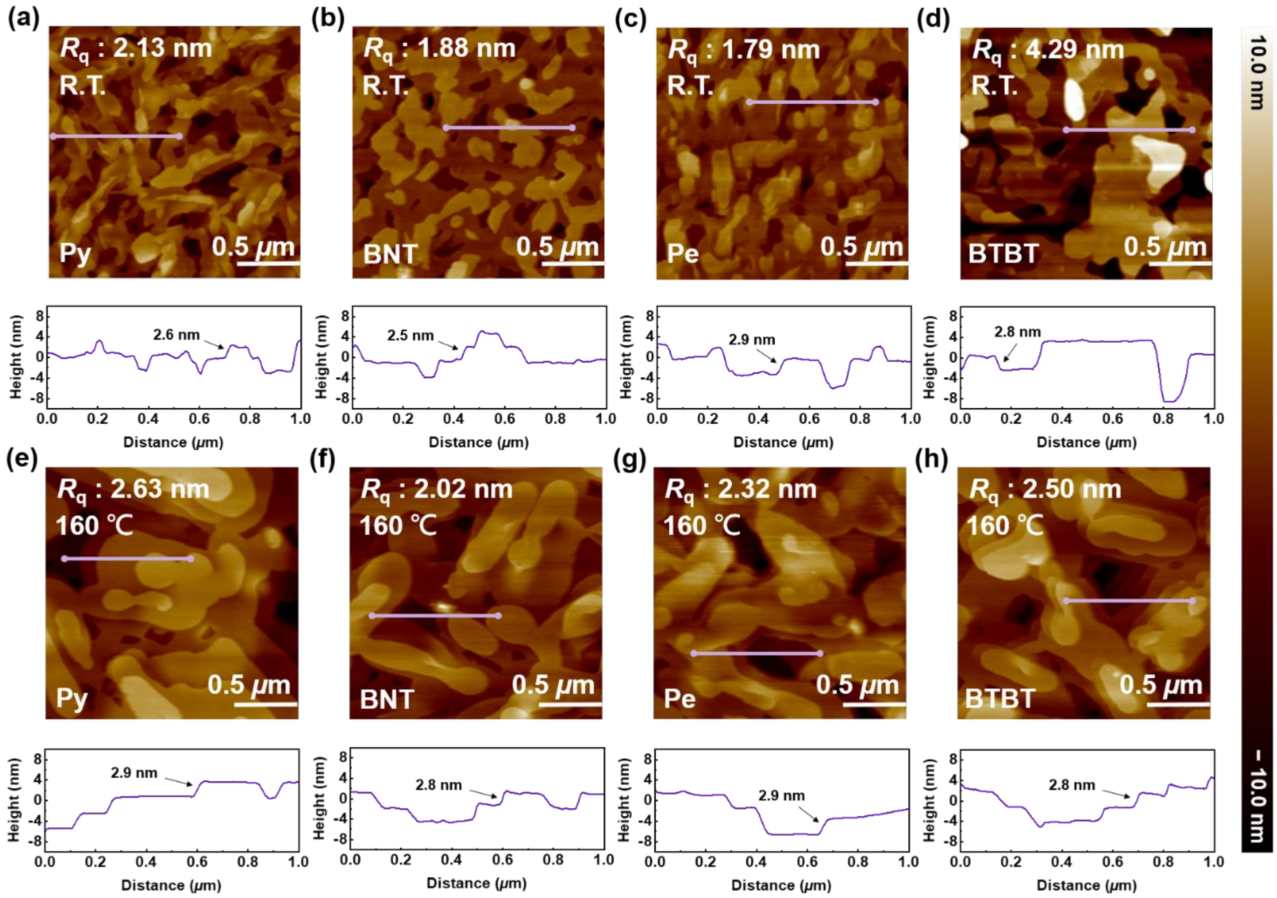

Figure S20. AFM height images and 1D topographic profiles of the Ph-BTBT-C10 on (a,e) Py, (b,f) BNT, (c,g) Pe, and (d,h) BTBT deposited at (a–d) room temperature (R.T.) and (e–h) 160 °C.

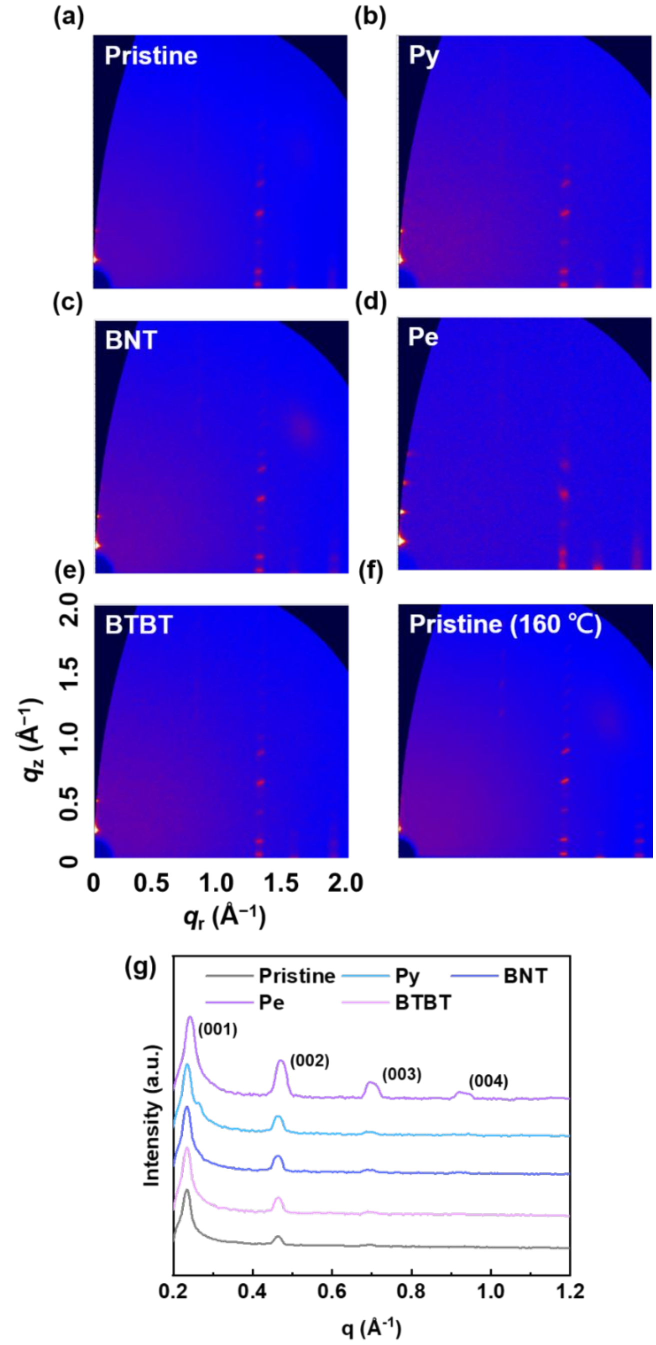

Figure S21. (a–f) 2D GIWAXS patterns of the Ph-BTBT-C10 deposited on different SAMs at (a–e) room temperature and (f) 160 °C. (g) 1D line-cutting profile in the out-of-plane direction of the Ph-BTBT-C10 deposited at room temperature onto different conjugated SAMs.

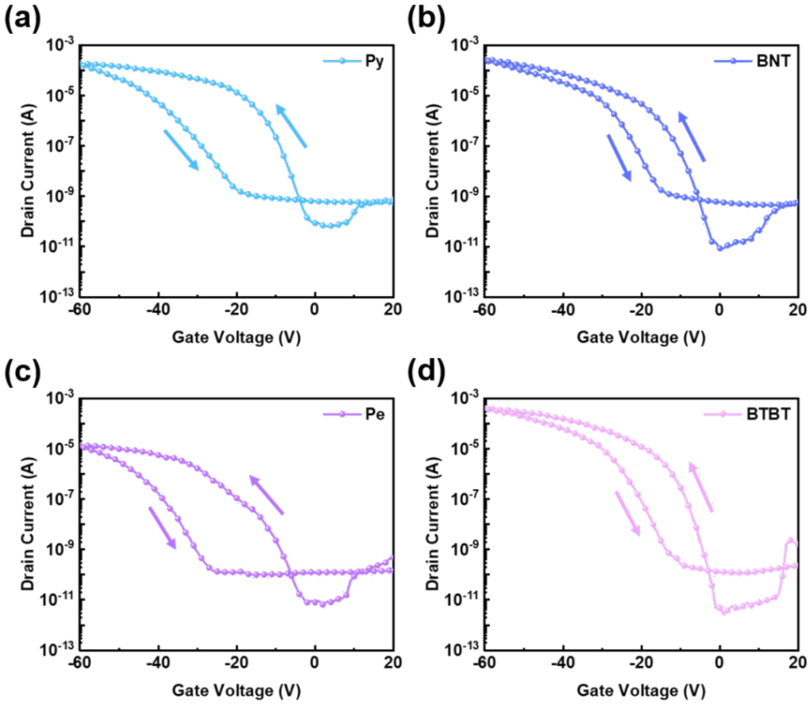

Figure S22. The dual sweep transfer curves of (a) Py, (b) BNT, (c) Pe, and (d) BTBT at $V_d = -50$ V.

Transfer characteristics.

The transfer characteristics were conducted with gate voltage (V_g) sweeping from +20 to −60 V under a drain voltage (V_d) of −50 V. When $V_g = -60$ V was applied for 1 s, the threshold voltage (V_{th}) presents a negative shift for all the conjugated SAM-based devices, which is considered as the writing process. In contrast, the erasing process was operated using 365-nm light (5.61 mW/cm^2) for 30 s under $V_d = -50$ V, showing a V_{th} shift toward the positive direction. The corresponding ($V_{th,write}$, $V_{th,erase}$) for Py, BNT, Pe, and BTBT are (−16.3, −2.5) V, (−20.5, −2.7) V, (−16.1, −6.0) V, and (−19.3, −3.7) V, respectively. The memory window (ΔV_{th}) is also defined as the V_{th} difference between the writing and erasing processes, representing the data storage capability for different states. The ΔV_{th} of 13.8, 17.8, 10.1, and 15.7 V were estimated for Py, BNT, Pe, and BTBT, respectively.

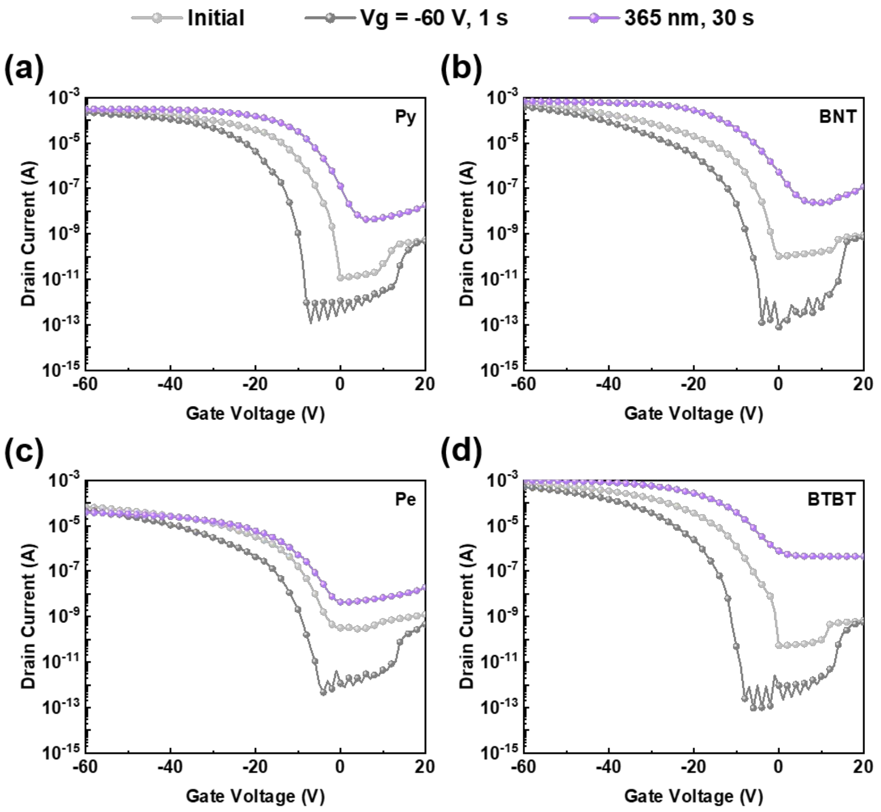

Figure S23. Transfer characteristics of (a) Py, (b) BNT, (c) Pe, and (d) BTBT under the illumination of 365-nm light (5.61 mW/cm^2) at $V_d = -50$ V.

Memory mechanism.

Since the semiconducting layer and the conjugated SAM layers all absorb UV light, the excitons can form in both layers, and the mechanisms were proposed separately. When a negative V_g was applied to the device, the induced holes were transferred to the SAM and stored as the trapped charges. First, in the semiconducting layer, the following light illumination produces the excitons in the channel, and the photogenerated electrons are moved to the SAM to recombine with the trapped holes. On the other hand, when the SAMs absorbed the light, the photogenerated excitons formed in the SAM, and the corresponding electrons recombined with the trapped charges. The holes were transferred to the semiconducting layer, producing an enhanced photocurrent during light illumination. After prolonged illumination, the excess electrons were further stored in the charge-trapping layer, leading to the bistable charge storage. Finally, the device returned to its initial state after a long duration. The proposed mechanism underlines the importance of the interaction between the interfaces.

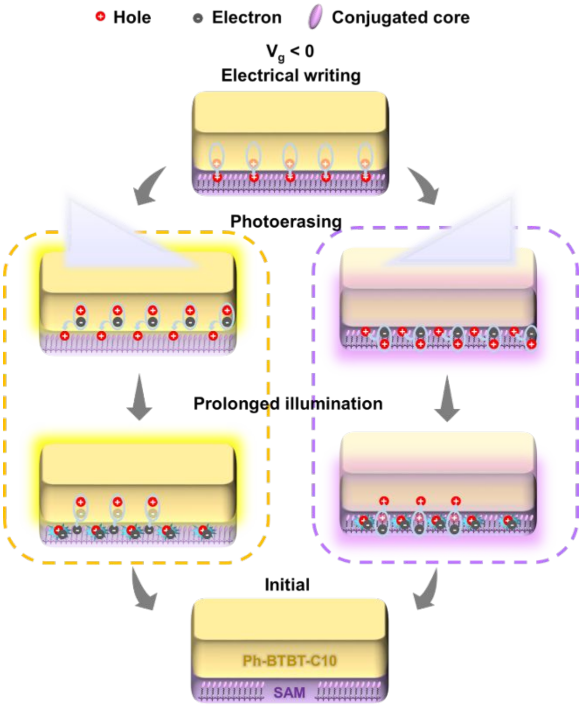

Figure S24. Schematic diagram of device working mechanism regarding the device architecture comprising semiconducting Ph-BTBT-C10 channel and SAM layers.

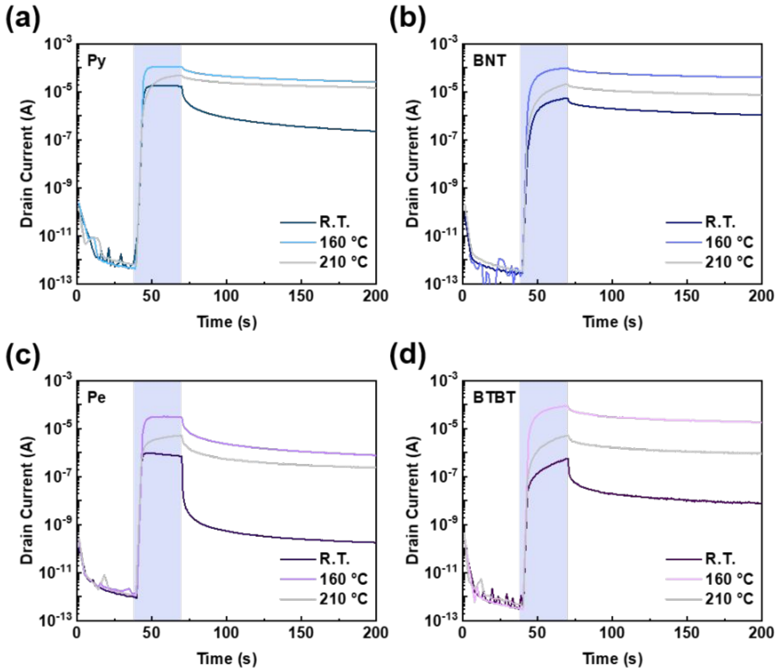

Figure S25. Transient characteristics of (a) Py, (b) BNT, (c) Pe, and (d) BTBT under 365 nm-light illumination for 30 s at V_d = −50 V and V_g = 0 V with Ph-BTBT-C10 deposited under different temperatures.

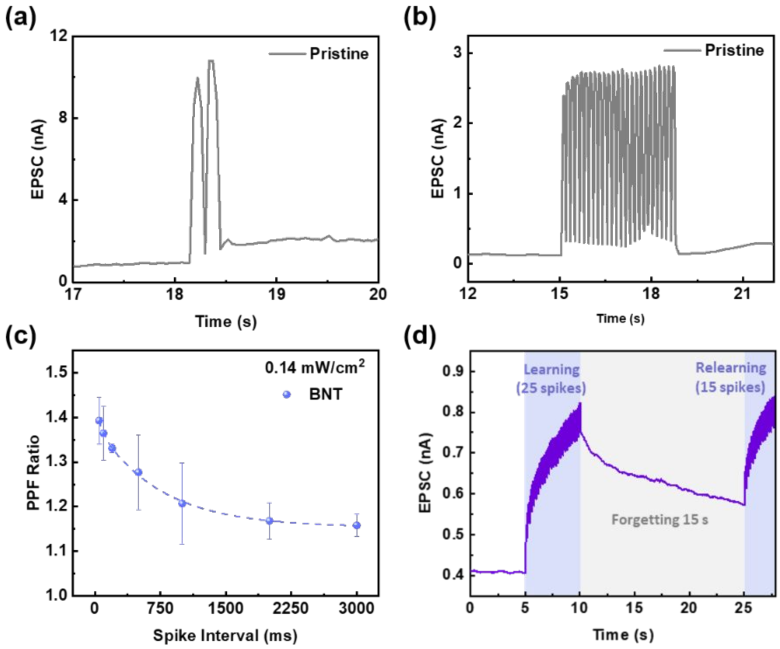

Figure S26. (a) EPSC variation to consecutive spikes of the pristine device. (b) Measurement of STM−LTM transition based on pristine device. (c) The relationship between PPF ratios and spike intervals. (d) The learning–forgetting–relearning behaviors in the human brain using a BNT-based device. Note that the operating conditions are 365-nm light illumination (spike width = 100 ms; spike interval = 50 ms) with light intensities of (a,d) 5.61 mW/cm^2 and (b) 0.14 mW/cm^2 and (c) 365-nm light illumination (spike width = 100 ms; 0.14 mW/cm^2) at V_d = −3 V, and the electrical properties were measured with a reading voltage of V_g = 0 V.

Device miniaturization using a thin Ph-BTBT-C10 channel.

A photosynaptic transistor with a semiconducting layer reduced to 20 nm was further fabricated to elucidate the concept of device miniaturization. Under light illumination (365 nm; 5.61 mW/cm^2) for 30s at V_d = −50 V, the scaled-down device based on BNT still retains a high current stability of 10^7–10^8 in LTM behavior (Figure S27a). In addition, a PPF ratio of 147% was achieved under two consecutive spikes (365 nm; 5.61 mW/cm^2; spike width = 100 ms) at V_d = −3 V (Figure S27b), which is comparable to the original device with thicker (50 nm) semiconducting layer. Finally, the repeated learning for the transition from STM to LTM was demonstrated by manipulating the spike number from 25 to 150, as shown in Figure S27c, Supporting Information. Therefore, the outstanding synaptic features, including STM, LTM, and the transition between these two behaviors, can be maintained in the BNT-based photosynaptic transistor with device miniaturization. Thus, the structural design involving the introduction of conjugated cores on the top of the carbon chains and proper molecular alignment, especially in the BNT molecule, contributes to the interplay between the two layers, giving rise to favorable memory and synaptic behaviors.

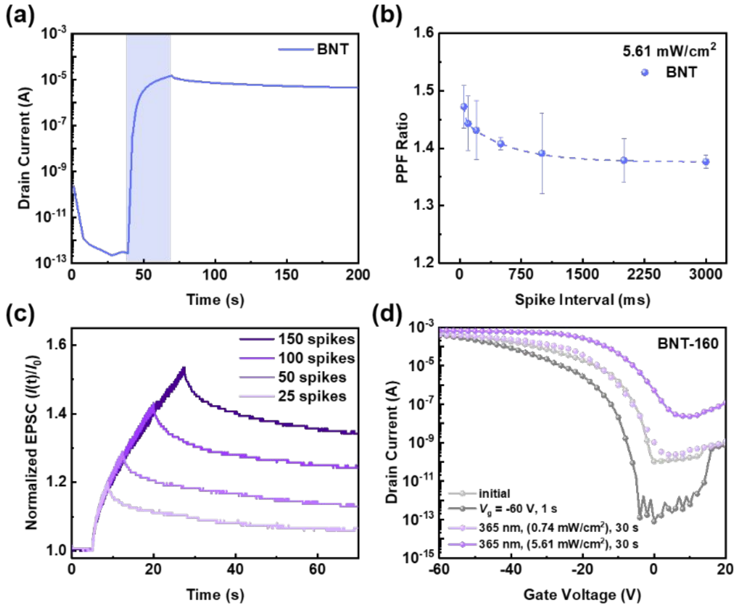

Figure S27. Electrical performances of the miniaturized photosynaptic transistor device based on BNT with a reduced channel thickness of 20 nm, including (a) transient characteristics under 365 nm-light illumination for 30 s at V_d = −50 V, (b) the relationship between PPF ratios and spike intervals under 365-nm light illumination (5.61 mW/cm^2) and a spike width of 100 ms at V_d = −3 V, and (c) emulation of human learning behavior and STM–LTM transition under 365-nm light illumination (0.14 mW/cm^2) at V_d = −3 V. (d) Transfer characteristics of BNT under the illumination of 365-nm light with intensities of 0.74 and 5.61 mW/cm^2 at V_d = −50 V. Note that the electrical properties were measured with a reading voltage of V_g = 0 V.

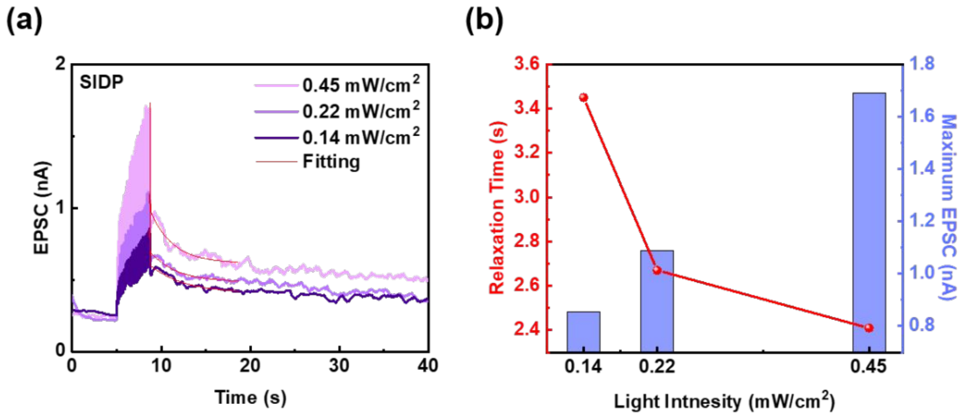

Figure S28. (a) STM–LTM transition of BNT by SIDP under 310-nm light illumination at $V_d = -3$ V and $V_g = 0$ V. (b) The corresponding relationship between relaxation time/maximum EPSC and spike intensity of BNT under 310-nm light illumination.

References

(1) Zuo, L.; Gu, Z.; Ye, T.; Fu, W.; Wu, G.; Li, H.; Chen, H. Enhanced Photovoltaic Performance of CH3NH3PbI3 Perovskite Solar Cells through Interfacial Engineering Using Self-Assembling Monolayer. *J. Am. Chem. Soc.* 2015, 137, 2674–2679.

(2) Kim, S. Y.; Kang, H.; Chang, K.; Yoon, H. J., Case Studies on Structure–Property Relations in Perovskite Light-Emitting Diodes via Interfacial Engineering with Self-Assembled Monolayers. *ACS Appl. Mater. Interfaces* 2021, 13, 31236–31247.

(3) Zhong, W.; Yao, R.; Liu, Y.; Lan, L.; Chen, R., Effect of Self-Assembled Monolayers (SAMs) as Surface Passivation on the Flexible a-InSnZnO Thin-Film Transistors. *IEEE Trans. Electron Devices* 2020, 67, 3157–3162.

(4) Tseng, C.-W.; Huang, D.-C.; Tao, Y.-T. Organic Transistor Memory with a Charge Storage Molecular Double-Floating-Gate Monolayer. *ACS Appl. Mater. Interfaces* 2015, 7, 9767–9775.

(5) Zheng, C.; Tong, T.; Hu, Y.; Gu, Y.; Wu, H.; Wu, D.; Meng, H.; Yi, M.; Ma, J.; Gao, D. Charge-Storage Aromatic Amino Compounds for Nonvolatile Organic Transistor Memory Devices. *Small* 2018, 14, 1800756.

(6) Chang, A. C.; Wu, Y. S.; Chen, W. C.; Weng, Y. H.; Lin, B. H.; Chueh, C. C.; Lin, Y. C.; Chen, W. C. Modulating the Photoresponsivity of Perovskite Photodetectors through Interfacial Engineering of Self-Assembled Monolayers. *Adv. Opt. Mater.* 2024, 12, 2301789.

(7) Sung, C. Y.; Chen, W. C.; Liu, C. L.; Lin, B. H.; Lin, Y. C.; Chen, W. C. Ultrafast Quasi-2D/3D Perovskite Photodetectors Conferred Using Interfacial Engineering of Self-Assembled Monolayers. *Adv. Opt. Mater.* 2024, 12, 2303241.

(8) Zhang, Y.; Liu, L.; Tu, B.; Cui, B.; Guo, J.; Zhao, X.; Wang, J.; Yan, Y. An Artificial Synapse Based on Molecular Junctions. *Nat. Commun.* 2023, 14, 247.

(9) Wu, Y. S.; Chang, A. C.; Chen, W. C.; Ercan, E.; Weng, Y. H.; Lin, B. H.; Liu, C. L.; Lin, Y. C.; Chen, W. C. High-Performance Synaptic Phototransistor Using a Photoactive Self-Assembled Layer toward Ultralow Energy Consumption. *Adv. Opt. Mater.* 2024, 12, 2302040.

(10) Guo, H.; Guo, J.; Wang, Y.; Wang, H.; Cheng, S.; Wang, Z.; Miao, Q.; Xu, X., An Organic Optoelectronic Synapse with Multilevel Memory Enabled by Gate Modulation. *ACS Appl. Mater. Interfaces* **2024**, https://doi.org/10.1021/acsami.3c19624.
